# Efficacy and safety of a food supplement with standardized menthol, limonene, and gingerol content in patients with irritable bowel syndrome: A double-blind, randomized, placebo-controlled trial

**DOI:** 10.1371/journal.pone.0263880

**Published:** 2022-06-15

**Authors:** Vladimir T. Ivashkin, Anna V. Kudryavtseva, George S. Krasnov, Yuri M. Poluektov, Margarita A. Morozova, Oleg S. Shifrin, Allan G. Beniashvili, Zarina A. Mamieva, Alexandra L. Kovaleva, Anatoly I. Ulyanin, Elizaveta A. Trush, Alexander G. Erlykin, Elena A. Poluektova

**Affiliations:** 1 I.M. Sechenov First Moscow State Medical University (Sechenov University), Moscow, Russian Federation; 2 Engelhardt Institute of Molecular Biology, Russian Academy of Sciences, Moscow, Russian Federation; 3 Mental Health Research Center, Moscow, Russian Federation; 4 Moscow State Linguistic University, Moscow, Russian Federation; Oregon State University, UNITED STATES

## Abstract

**Background:**

Irritable bowel syndrome (IBS) affects 9,2% of the global population and places a considerable burden on healthcare systems. Most medications for treating IBS, including spasmolytics, laxatives, and antidiarrheals, have low efficacy. Effective and safe therapeutic treatments have yet to be developed for IBS.

**Purpose:**

This study assessed the efficacy and safety of a food supplement containing standardized menthol, limonene, and gingerol in human participants with IBS or IBS/functional dyspepsia (FD).

**Design:**

A double-blind, randomized, placebo-controlled trial.

**Methods:**

We randomly assigned 56 patients with IBS or IBS/FD to an intervention group (Group 1) or control group (Group 2) that were given supplement or placebo, respectively, in addition to the standard treatment regimen for 30 d. Three outpatient visits were conducted during the study. Symptom severity was measured at each visit using a 7×7 questionnaire. Qualitative and quantitative composition of the intestinal microbiota were assessed at visits 1 and 3 based on 16S rRNA gene sequencing.

**Results:**

At visit 1 (before treatment), the median total 7×7 questionnaire score was in the moderately ill range for both groups, with no difference between the groups (*p* = 0.1). At visit 2, the total 7×7 score decreased to mildly ill, with no difference between the groups (*p* = 0.4). At visit 3, the total score for group 1 indicated borderline illness and for group 2 remained indicated mild illness (*p* = 0.009). Even though we observed some variations in gut microbiota between the groups, we did not find any statistically significant changes.

**Conclusion:**

The food supplement with standardized menthol, limonene, and gingerol content increased the efficacy of standard therapy in IBS and FD patients. The use of the supplement did not cause any obvious side effects.

**Registration:**

ClinicalTrials.gov Identifier: NCT04484467

## Introduction

Irritable bowel syndrome (IBS) is the most prevalent functional disorder worldwide and 27.0%–82.6% of patients with IBS exhibit symptoms of functional dyspepsia (FD) [1, 2]. These diseases impair the patient’s quality of life, reduce their capacity for social activity, and create significant healthcare expenses [3]. Based on diagnosis by Rome III criteria, the prevalence of IBS is estimated at 10,7% in Bangladesh and 9,2% worldwide [4].

Most medications for IBS, including spasmolytics, laxatives, and antidiarrheals, have relatively low efficacy and optimal treatment approaches for IBS and FD have yet to be developed [5, 6]. Tricyclic antidepressants (TCAs) and selective serotonin reuptake inhibitors have shown superior efficacy in the treatment of IBS and FD in placebo control studies, though adverse effects have a high prevalence, particularly among patients treated with TCAs [7, 8].

Recently, a relationship was established between gut microbiota and a functional gastrointestinal tract disorder [9]. The composition of the intestinal microbiota in patients suffering from IBS is characterized by decreased content of bacterial cells producing short-chain fatty acids (SCFAs) [10], which regulates the expression of tight contact proteins, T-lymphocytes, and cytokines and ensures adequate permeability of the intestinal barrier [11]. Disruption of intestinal barrier permeability leads to inflammatory responses, changes in sensitivity and motility, and the development of disease symptoms in the intestinal wall [12–14]. Therefore, the inclusion of probiotics in the treatment regimen promotes overall therapeutic efficacy [15, 16].

IBS pathogenesis represents a complex interplay among changes in intestinal permeability and microbial dysbiosis within the gut, altered mucosal immune function, visceral hypersensitivity, impaired gut motility [17]. Given the comprehensively described pathology of IBS, we aimed to analyze the efficacy of treatment with a food supplement and how its components affect each pathogenetic stage as well as the composition of intestinal microbiota.

Some herbal ingredients have been reported to relieve IBS symptoms. Menthol has an antispasmodic, analgesic effect and suppresses the growth of pathogenic microorganisms; D-limonene participates in esophagus and stomach mucus barrier recovery; and gingerol has an antispasmodic and prokinetic effect (Table 1). Thus, the proposed treatment consists of these components and should cover almost all known pathophysiological links of IBS disorder.

**Table 1:** Components of the supplement and their mechanisms of action.

Component	Antispasmodic effect	Analgesic effect	Effect on the intestinal microbiota composition	Prokinetic effect	Esophagus and stomach mucus barrier recovery
Menthol	**+** Calcium channel blocking [18] TRPM8 channel receptor activation [18] Nicotinic cholinergic receptor desensitization [18]	**+** Blocking of central [19] and peripheral Na+ channels [20] GABA receptor activation [20]	**+** Inhibition of pathogenic microorganisms: *H*. *pylori*, *S*. *enteritidis*, *E*. *coli 0157*:*H7*, *and S*. *aureus* [21]	**-**	**-**
D-limonene	**-**	**-**	-	**+** [22]	**+** Decrease of NO and prostaglandin E2 expression [23, 24] Decrease of TNF-α and IL-6 levels in plasma [23]
Gingerol	**+** Suppression of Ca^2^+ entry into cells via L-type Ca^2+^ channels and inhibition of ionotropic 5-HT-3 receptors [25]	**-**	**-**	**+** Suppression of 5-HT-3 and 5-HT-4 receptors [26] Activation of M3 cholinergic receptors in the stomach [27] Inhibition of M1 and M2 presynaptic cholinergic receptors [27]	**-**

* The mechanism is not studied, yet.

We designed a double-blind, randomized, placebo-controlled trial to assess the efficacy and safety of a food supplement standardized for menthol, limonene, and gingerol content (hereinafter referred to as “the supplement”) in IBS and IBS/FD patients.

## Methods

### Trial design

We designed an interventional (clinical trial), randomized, double-blind controlled trial protocol that was approved by the Ethics Committee of the Mental Health Research Center, Moscow, Russian Federation (no. 418, 01/31/2018). Written informed consent was obtained from all participants.

### Participants

**Fig 1:**
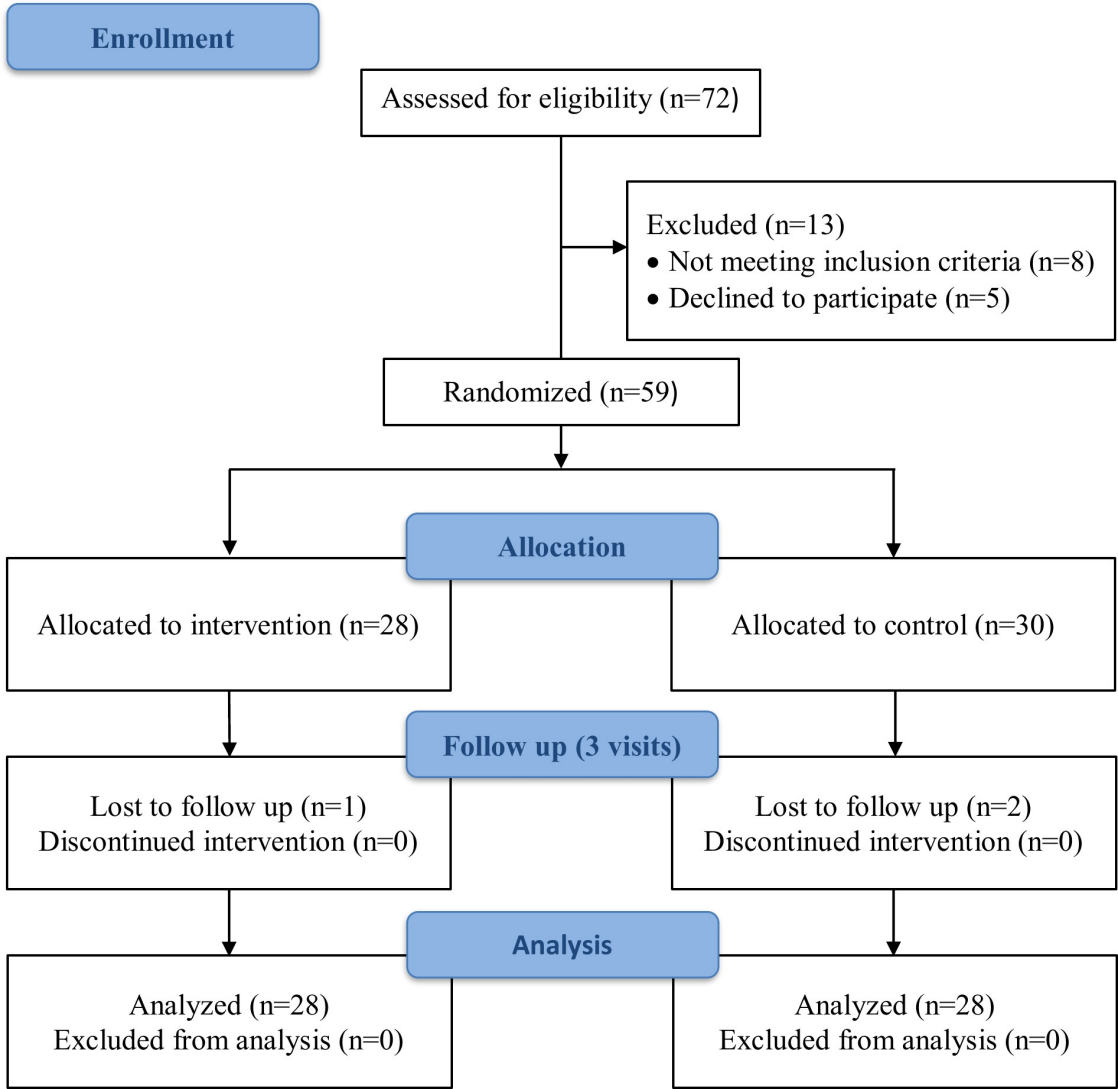
CONSORT flow diagram.

Participants with IBS or IBS and FD were recruited during their outpatient medical treatment at the Mental Health Research Center and Sechenov University, Moscow, Russia. Symptoms were validated according to the Rome IV criteria. Non-functional causes for the symptoms were excluded after a detailed evaluation of medical history, physical examination, extensive panel of blood tests, stool analysis, and colonoscopy with biopsies [28, 29]. The exclusion criteria were: patients younger than 18 years, older than 59 years, with organic bowel disease, with renal disease, with hepatic insufficiency, or with mental illness significantly impairing self-report (e.g., schizophrenia, bipolar disorder, or epilepsy) (Fig 1).

### Interventions

#### Standard treatment regimen

Diarrhea-predominant IBS patients (IBS-D) and mixed bowel habits IBS patients (IBS-M) were treated with spasmolytics. Constipation-predominant IBS patients (IBS-C) were treated with spasmolytics and laxatives. IBS/FD patients were treated with spasmolytics and proton pump inhibitors.

#### Additional treatment regimen

At visit 1, all patients were randomly assigned into two groups and were assigned a 30-day standard treatment regimen and 1 capsule, 730 mg, once a day in a double-blind manner. The intervention group (group 1) received the supplement “Standart Zdorovya GASTRO” and the control group (group 2) received a placebo (Table 2). During the study period, three outpatient visits were conducted (visit 1 at day 1, visit 2 at 15 ± 2 d, and visit 3 at 30 ± 2 d).

**Table 2:** Components in the supplement and placebo capsules.

Drug	Components	Amount of substance (mg)
Supplement “Standart Zdorovya GASTRO”	Peppermint oil (40% menthol, 1.5% limonene)	240
	Ginger oil (14% gingerol)	50
	Olive oil	440
Placebo	Olive oil	730

### Outcomes

#### Primary outcome measures

Changes in symptom severity of IBS and FD (constipation-predominant, diarrhea-predominant, mixed-type IBS and IBS/FD) were assessed at each visit using a 7×7 questionnaire.

#### Secondary outcome measures

Changes in the number of SCFA producing bacteria as well as the qualitative and quantitative composition of intestinal microbiota were assessed at visits 1 and 3, based on 16S rRNA gene sequencing data.

### Sample size

Due to a lack of comparable studies, we were unable to determine an appropriate sample size. Using GPower 3.1 software, a sample size of 38 patients was calculated according to Jacob Cohen’s effect size estimation method: statistical significance *α* = 0.05, power (1-*β*) = 0.80, and expected effect size *d* = 0.70 (moderate to strong) [30]. To increase the statistical power of the study, a total of 56 patients were recruited.

### Randomization

According to symptom severity, the patients were assigned to one of six groups, determined by the sum of the 7×7 scores: 0–1, normal (healthy); 2–6, borderline ill; 7–12, mildly ill; 13–18, moderately ill; 19–24, markedly ill; >25, severely ill. Effective therapy of IBS greatly decreased the sum of the 7×7 scores (by more than 6 points) in previous studies [31] and effect size (Cohen`s *d*) can reach 2. The effect size values normally range from 0.3 (low effectiveness of treatment) to 0.5 (moderate effectiveness) and up to 0.8 (high effectiveness). We assumed that the use of food supplements would show a high effect size and reduced the overall 7×7 scores. Thus, clinically significant decreases in the total 7×7 score (5.4 points on average) should have an effect size of 0.07.

### Blinding

Being a double-blind trial, both participants and investigators were blinded to the treatment. Placebo and food supplement “Standart Zdorovya GASTRO” were delivered to the center in non-transparent packages. The containers were numbered by the manufacturer and neither the researchers nor the patients knew what number belonged to placebo or food supplement “Standart Zdorovya GASTRO”.

### Assessment tools

At each visit, patients completed the 7×7 questionnaire [32] as an assessment of symptom severity. At visits 1 and 3, the qualitative and quantitative composition of intestinal microbiota were determined based on 16S rRNA gene sequencing.

### 7×7 Questionnaire

The 7×7 questionnaire allows patients to record the main symptoms of FD and IBS and the physician to obtain a quantitative description of symptom frequency and intensity for objective evaluation. The following points are assigned according to symptom frequency: none, 0 points; once a week, 1 point; two to three times a week, 2 points; daily, 3 points; and several times a day, 4 points. The intensity is estimated as mild (1 point), moderate (2 points), and severe (3 points). Solid/“nuts” stool once a week and two to three times a week are scored at 5 points and 2 points, respectively. Liquid or mushy stool with other symptoms (pain in the stomach area, a feeling of burning in the stomach area, fullness in the stomach, early satiety, abdominal pain that decreases after a bowel movement, or bloating) is assigned 0 to 4 points. The scores reflecting symptom presence and intensity are summarized, and the scores obtained for each symptom are summed. According to the total score, patients would be assigned to one of six groups: 0–1 points, healthy; 2–6 points, borderline ill; 7–12 points, mildly ill; 13–18 points, moderately ill; 19–24 points, markedly ill; and 25 or more points, severely ill [19].

### Safety

The safety of treatment was assessed at Visits 1, 2 and 3 by interviewing the patients about the additional complaints. Blood samples were collected for the routine blood tests (hemoglobin level, red blood cell count, white blood cells count, erythrocyte sedimentation rate, aspartate aminotransferase level, alanine aminotransferase level, alkaline phosphatase level, gamma-glutamyltransferase level, creatinine level, pancreatic alpha-amylase level) at visit 1 and 3.

### DNA isolation, 16S library preparation, and qualitative and quantitative composition of intestinal microbiota based on 16S rRNA gene sequencing

Total DNA was isolated with an AmpliPrimeDNA-sorb-AM kit (NextBio, Moscow, Russia) for clinical specimens, according to the manufacturer’s protocol, and stored at –20°C. For qualitative and quantitative assessment of the isolated DNA, we used a NanoDrop 1000 spectrophotometer (Thermo Fisher Scientific, Waltham, MA, USA). Next, a 16S library was prepared according to the Illumina MiSeq protocol for 16S Metagenomic Sequencing Library Preparation (Illumina, San Diego, CA, USA). The first round of 16S rDNA amplification of the V3–V4 variable regions was performed using the following primers: forward 5′-TCGTCGGCAGCGTCAGATGTGTATAAGAGACAG-CCTACGGGNGGCWGCAG-3′ and reverse 5′-GTCTCGTGGGCTCGGAGATGTGTATAAGAGACAG-GACTACHVGGGTATCTAATCC-3′. These primers are aimed at the amplification of bacterial (more than 90% taxonomic coverage) but not archaeal (less than 5%) rRNA genes. The amplification cycle (2720 Thermal Cycler, Applied Biosystems, Waltham, MA, USA) was programmed as: 1) 95°C for 3 min; 2) 30 cycles of 95°C for 30 s, 55°C for 30 s, and 72°C for 30 s; 3) 72°C for 5 min; and 4) 4°C.

The derived amplicons were purified using Agencourt AMPure XP beads (Beckman Coulter, Brea, CA, USA) according to the manufacturer’s protocol. The second amplification round was used to double-index the samples with a combination of specific primers. The amplification program was set to: 1) 95°C for 3 min; 2) 8 cycles of 95°C for 30 s, 55°C for 30 s, and 72°C for 30 s; 3) 72°C for 5 min; and 4) 4°C.

The products of the second PCR round were purified using Agencourt AMPure XP beads. The concentrations of the 16S rDNA libraries were measured with a Qubit 2.0 fluorimeter (Invitrogen, Waltham, MA, USA) utilizing a Quant-iT dsDNA High-Sensitivity Assay Kit. The purified amplicons were mixed equimolarly according to the concentration values. The quality of the libraries was evaluated using an Agilent 2100 Bioanalyzer (Agilent Technologies, Santa Clara, CA, USA) and an Agilent DNA 1000 Kit. Sequencing was carried out on a MiSeq system (Illumina) with a MiSeq Reagent Kit v2 (paired-end reads, 2 × 250 nt, Illumina).

### The analysis of 16S rRNA gene sequencing data

Since the reads overlap was small (the average amplicon length was about 440–460 bp at sequencing 2 × 250 nt), the reads were pre-merged (before analysis, in DADA2) using the MeFiT tool [33]. For most samples, more than 99.5% of the reads were successfully merged and analyzed by the DADA2 package for R [34]. For the analysis: 1) primer sequences were removed using cutadapt; 2) the reads were filtered by quality; 3) error distribution models were derived based on read quality profiles; 4) sequencing errors were corrected; 5) ribosomal sequence variants (RSV), OTU analogues, were inferred; and 6) chimeric RSVs, accounting for 62% and 4.6% of all RSV and all reads, respectively, were eliminated. Next, taxonomic annotation of the RSVs (7,551 after the removal of chimeras) was carried out using DADA and the 16S RDP reference sequence database [35]. The data obtained were also analyzed utilizing the Piphillin web service [36], which predicts the metabolic potential of a bacterial community based on the analysis of representative 16S rRNA gene sequences (OTU or RSV). Further analysis was carried out using our own R (including the vegan, fossil, ggplot2, and pheatmap packages) and Python scripts. A Mann-Whitney test was used for comparison between groups, a Wilcoxon test for comparison between visits to the same patient, and a Student’s *t*-test for comparison of normally distributed data. Spearman correlation coefficients were calculated.

### Statistical analysis

Statistical analysis was carried out using standard functions and additional packages in the R environment [37, 38]. To assess differences between groups of patients, analysis of contingency tables (including Fisher’s exact test) and a non-parametric Mann-Whitney test were used. The differences between visits in each group of patients was determined using a Friedman Rank test and a post-hoc Wilcoxon signed-rank test for paired samples, with p-values adjusted according to Bonferroni multiple testing (FDR). The null hypothesis assumed the absence of a relationship between the type of therapy/stage of the study and the change in the analyzed parameter. Two-sided p-values <0.05 were considered statistically significant.

### Deviations from the original protocol

There were no deviation from the original protocol.

### Study

As this was originally a pilot study, we assumed that the data would not be sufficient for a full-scale clinical trial or publication. Thus, the study was registered only after the initiation of patient recruitment. The authors can confirm that all ongoing and related trials have been registered.

## Results

The trial was performed from February 2018 to December 2019 at the Mental Health Research Center, Moscow, Russia. Two groups of 56 patients collectively completed the study (Table 3). There were no significant differences in gender, age, diagnosis, disease duration, and 7×7 questionnaire results between the groups at the first visit.

**Table 3:** Comparison of patients in groups 1 and 2 by sex, age, and disease duration.

Sign	Group 1 (intervention) (*n* = 28)	Group 2 (control) (*n* = 28)	*p-value*
Age, years	35.25 (±11.84)	31.8 (±10.76)	0.30
Sex, n (%)			
Men	13 (46%)	10 (36%)	0.79
Women	15 (54%)	18 (64%)	
Diagnosis, n (%)			
IBS	20 (71%)	19 (68%)	0.17
IBS+FD	8 (29%)	9 (32%)	
IBS duration, months*	48 (17.5–111)	48 (24–90)	0.98
Total 7×7 questionnaire score**	13.2 (11.4–15.0)	15.9 (13.4–18.5)	0.1

*- Median (1–3 quartiles, Q25-Q75);

**- 95% Confidence interval (CI).

### 7×7 Questionnaire

**Fig 2:**
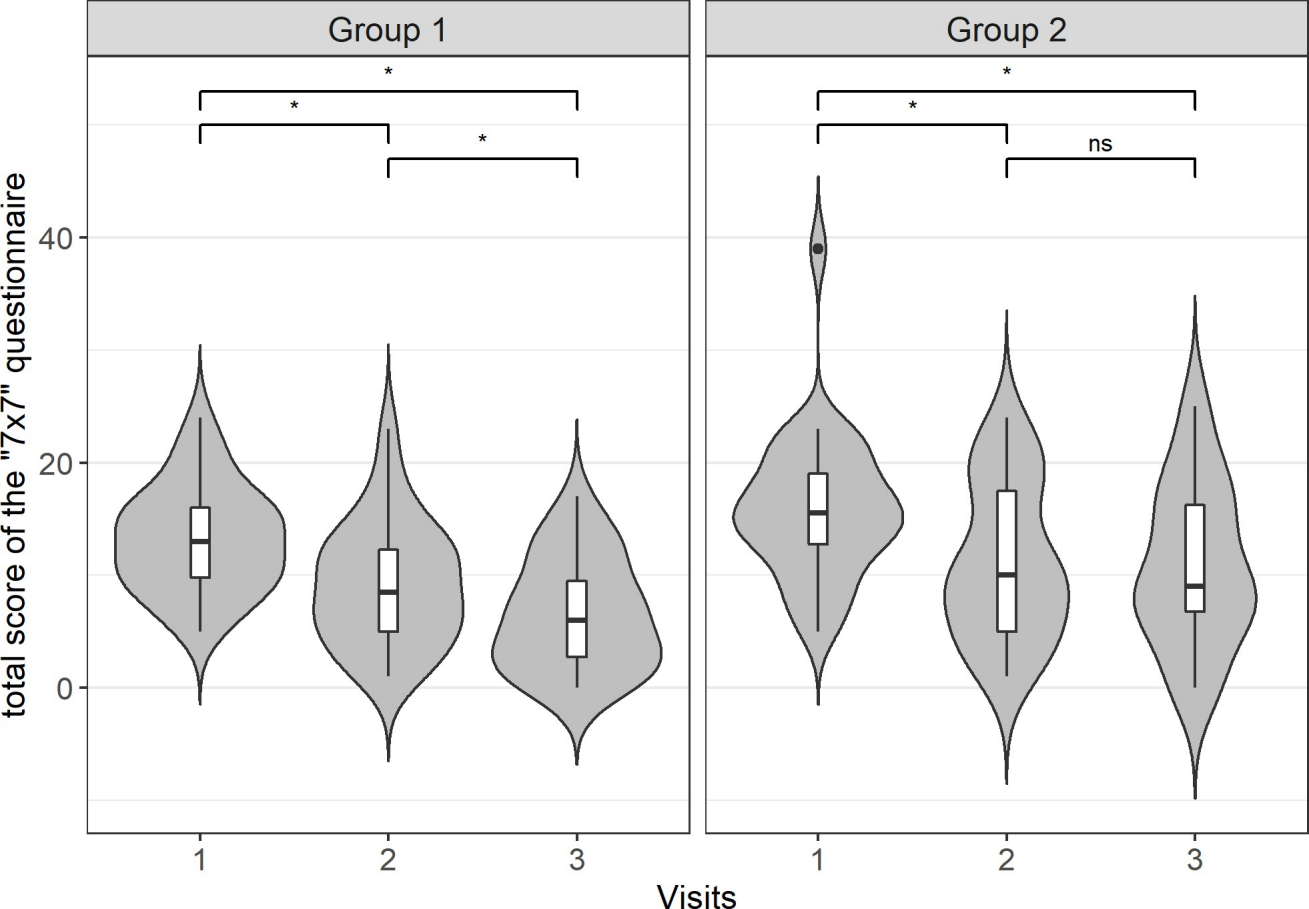
Total 7×7 questionnaire scores in group 1 and group 2 at visits 1, 2, and 3.

There was no statistically significant difference in the total 7×7 questionnaire scores between the groups at visit 1 and patients in both groups were moderately ill (see Table 2). Throughout all three visits, the treatment effect on the total 7×7 questionnaire scores was tested using the Friedman test (Table 1 in S4 File) followed by the Wilcoxon signed-rank test (Table 2 in S4 File). There was a statistically significant decrease in the total 7×7 questionnaire score in both groups to scores indicating mild illness at visit 2 (mean difference, –4.04; 95% CI, –6.10 to –1.97; *p* = 0.001 in group 1 and mean difference, –4.86; 95% CI, –6.75 to –2.96; *p* = 0.0004 in group 2). There were no significant differences (*p* = 0.42) between groups at visit 2. At visit 3, the total score in group 1 decreased to a score indicating borderline illness (mean difference, –2.68; 95% CI, –4.29 to –1.07; *p* = 0.009). Group 2 remained in the score range indicating mild illness (Fig 2).

The total 7×7 questionnaire score decreased significantly in group 1 from 13 (95% CI 11.4–15.0) at visit 1 to 9.18 (95% CI 7.07–11.3) at visit 2 and 6.5 (95% CI 4.58–8.42) at visit 3. In group 2, the 7×7 questionnaire score decreased significantly between visits 1 and 2 (15.9 [95% CI 13.4–18.5] to 11.1 [95% CI 8.4–13.7]). * The differences between the groups are significant (*p* < 0.05); ns–non-significant.

Between visit 1 to visit 3, there was a decrease in the number of group 1 patients with epigastric pain (*p* = 0.029), solid/”nuts” stool (*p* = 0.005), abdominal pain that decreases after a bowel movement (*p* < 0.0001), and bloating (*p* = 0.015) (S1–S6 Figs in S3 File).

#### Qualitative and quantitative composition of the intestinal microbiota based on 16S rRNA gene sequencing

**Fig 3:**
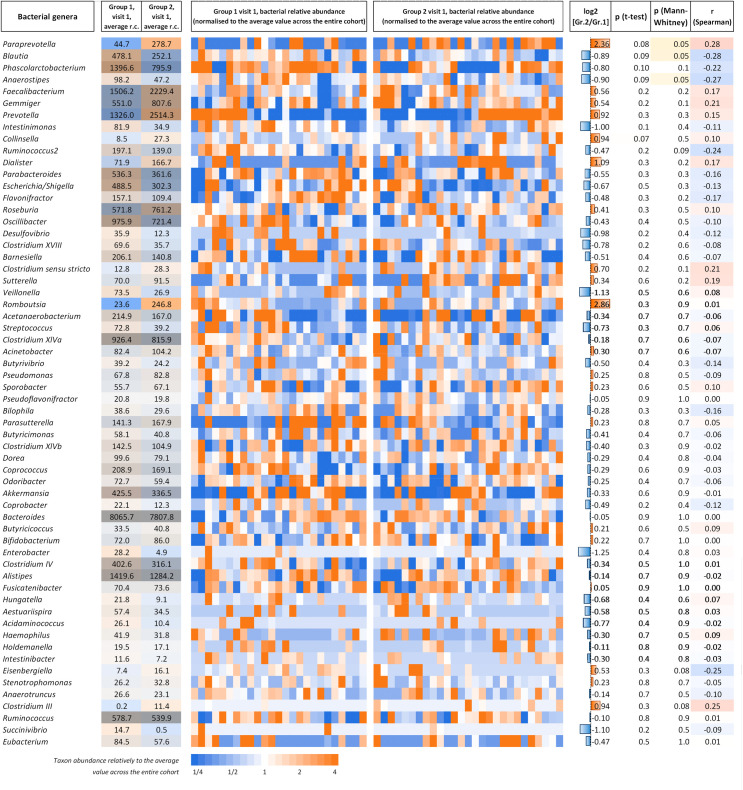
Qualitative and quantitative composition of the intestinal microbiota genera in patients of groups 1 and 2 at visit 1. Columns 2 and 3 present the relative bacterial content of a given genus (r.c., represents the normalized number of reads annotated to the current genus) averaged over all samples of a group. The color brightness reflects the average content, whereas the color hue (blue to orange) indicates the difference between the groups. The heatmaps in columns 4 and 5 show the bacterial content profiles of the genera for individual samples. The genera are sorted by a scoring factor, which reflects the magnitude of differences between groups 1 and 2, statistical significance (based on the Mann-Whitney test), and the average bacterial content across all samples.

At visit 1, we observed some differences in the gut microbiota composition between groups 1 and 2. For several genera, we noticed 1.5–2.5-fold change in the average bacterial content (normalized read counts per taxon) between the groups, some of which passed the Mann-Whitney U-test *p* < 0.05 threshold, but none with statistically significant FDR (e.g., *Blautia*, *Anaerostipes*, and *Paraprevotella*). The 60 bacterial genera with the highest abundance in patients of groups 1 and 2 at visit 1 are presented in Fig 3. A high degree of heterogeneity was observed between patients.

**Fig 4:**
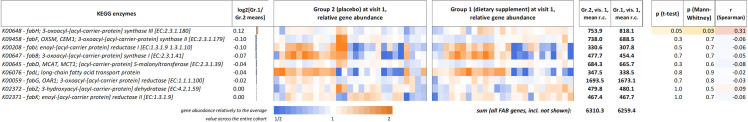
Relative content in the metagenome of genes involved in fatty acid biosynthesis (FAB) in patients of groups 1 and 2 at visit 1 (inferred from taxonomic data). Columns 5 and 6 show the average content of FAB genes in each group. The heatmaps in columns 3 and 4 demonstrate the relative gene abundance per sample. Samples are re-arranged according to the similarity of gene abundance profiles.

Using the Piphillin web service, we inferred the relative abundance of genes involved in fatty acid biosynthesis (FAB) at visit 1. While the total abundance of all genes involved in FAB was practically identical (differing by less than 1%), significant differences were noted for the *fabH* gene (Fig 4), though it did not pass the FDR threshold.

**Fig 5:**
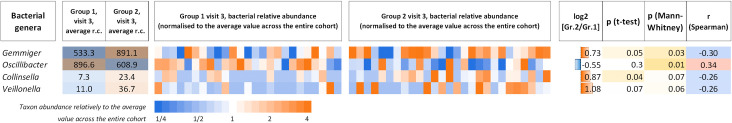
Statistically significant differences in the composition of intestinal microbiota genera between groups 1 and 2 at visit 3. Columns 2 and 3 present the relative content (r.c.) of bacterial genera averaged over all samples in each group. The heatmaps in columns 4 and 5 show the bacterial content profiles for individual samples.

At visit 3, we observed some differences in the composition of gut microbiota between groups 1 and 2. *Oscillibacter* had higher prevalence in group 1, whereas *Veillonella*, *Collinsella*, and *Gemmiger* were more prevalent in group 2 (Fig 5). However, the overall magnitude of these differences was comparable to that at visit 1. Although p-values (Mann-Whitney U-test) for these events were lower than 0.05, they did not pass the FDR < 0.05 threshold.

**Fig 6:**
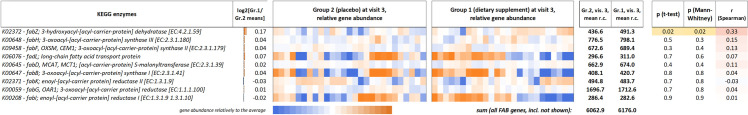
The relative content in the metagenome of genes participating in FAB in patients of groups 1 and 2 at visit 3 (inferred from taxonomic data). Columns 5 and 6 show the average content of FAB genes in each group. The heatmaps in columns 3 and 4 demonstrate the relative gene abundance per sample. The samples are re-arranged according to the similarity of gene abundance profiles.

The same observations were made for the relative content of genes involved in FAB (Fig 6). In this case, very slight (~2%) differences were noted for individual genes (*fabZ*) in favor of the patients taking dietary supplements. Even though they passed the Mann-Whitney U-test *p* < 0.05 threshold, they did not pass multiple test adjustments (FDR). The magnitude of inter-group differences at visit 3 was comparable to that at visit 1.

#### Correlation analysis between microbiome composition and 7×7 questionnaire scores

In order to identify the genera and families of bacteria associated with IBS severity, we carried out a correlation analysis between the relative abundance of various bacterial taxa and the sum of the 7×7 questionnaire scores across all visits. Statistically significant associations (that passed the FDR threshold < 0.05) were noticed only for the genus *Fusobacterium* and its related family *Fusobacteriaceae* (*r* = 0.29, *p* = 0.0002, FDR = 0.02). However, this family had a relatively low overall abundance (mean of 3 reads per 1 sample, found only in 11% of samples).

Additionally, we identified a number of families statistically associated with IBS severity, though they did not pass the FDR adjustment threshold. Among them were *Acidaminococcaceae* (Spearman’s *r* = –0.23, *p* = 0.005); *Enterobacteriaceae* (*r* = –0.16, *p* = 0.05); *Streptococcaceae*, *Coriobacteriaceae*, and *Veillonellaceae* (*r* = 0.18 … 0.21, *p* = 0.01 … 0.03). We noted positive correlations between *Butyrivibrio*, *Catenibacterium*, *Akkermansia*, *Alloprevotella*, *Dialister*, and *Prevotella* abundance with age (*r* = 0.17 … 0.24); inverse correlations of *Roseburia*, *Megasphaera*, *Bacteroides*, *Odoribacter*, *Parabacteroides*, *Alistipes*, and *Parasutterella* abundance with age (*r* = –0.17 … –0.28); and associations of *Roseburia*, *Mitsuokella*, *Coprobacter*, and *Escherichia*/*Shigella* abundance with gender.

Finally, we assessed the relationship between the metabolic potential of the collective microbiome (inferred from taxonomy) and disease severity (inferred from the 7×7 questionnaire score). Correlations were observed for 360 predicted bacterial genes, though none of them passed the FDR threshold. Interestingly, none of these genes were related to FAB. However, the 26^th^ place (out of 4,405 genes) was marked by the *atoE* gene encoding for a short-chain fatty acid transporter. The correlation coefficient of its relative abundance and 7×7 score was *r* = –0.23 (*p* = 0.004). When analyzing associations with age, we noted 1,088 or 186 predicted bacterial genes at a given threshold for *p* < 0.05 or FDR < 0.05.

#### Correlation analysis between microbiome composition and 7×7 questionnaire scores

The safety of the food supplement “Standart Zdorovya GASTRO” was measured according to additional complaints recorded during each visit. Also, blood tests were performed at visit 1 and 3, with no deviations from the reference values observed. In group 2 some changes were observed in sedimentation rate and red and white blood cell count, but these values did not exceed the reference ranges (S7–S16 Figs in S3 File). No additional complaints were recorded in either of the groups.

## Discussion

The development and maintenance of functional gastrointestinal disorders can be attributed to various psychological and biological factors. Changes in the composition of intestinal microbiota and intestinal permeability that decrease tight junction protein expression and reduce the thickness of the mucus layer are key factors impairing gastrointestinal motility and sensitivity [39–41].

Microbial SCFAs are vital to intestinal homeostasis. They suppress the growth of Gram-negative pathogens, function as energy sources for colonocytes, and have anti-inflammatory effects. SCFAs are organic fatty acids with 1–6 carbon atoms and are the principal anions arising from the bacterial fermentation of polysaccharides, oligosaccharides, proteins, peptides, and glycoprotein precursors in the colon [42]. The main SCFAs are produced by bacterial cells using acetyl-CoA by different pathways, such as acrylate and succinate [43].

We used the food supplement “Standart Zdorovya GASTRO” with standardized menthol, limonene, and gingerol content to deliver antispasmodic [18, 25], analgesic [19, 20], anti-inflammatory, and prokinetic [19, 20, 22, 25–27] effects. The supplement has also been shown to contribute to the restoration of intestinal microbiota [21] and gastrointestinal mucosal function [23, 24] (Table 1). Thus, the inclusion of this supplement in IBS or IBS/FD treatment regimens can improve the efficacy of standard therapy. Patients who received the supplement showed significant symptom reduction. A significant decrease in symptom severity to borderline ill was observed in group 1 at visit 3 (7×7 score –6.5, 95% CI 4.58–8.42), whereas group 2 remained mildly ill (total 7×7 score 10.8, 95% CI 8.00–13.6). Furthermore, the number of IBS and FD symptoms decreased in more patients of group 1 than group 2.

The greater treatment efficacy in group 1 (standard treatment regimen and dietary supplement) might be associated with modifications in the intestinal microbiome and metabolome. However, we generally observed heterogeneous taxonomic profiles of microbiome community structure between patients, both at visits 1 and 3. This complicates the interpretation of changes caused by the intake of the dietary supplement. Similarly, we found a very small increase in the content of specific genes involved in the biosynthesis of fatty acids at visit 3. However, here it is also impossible to confirm that the changes were due to the effects of the dietary supplement. Nevertheless, the analysis of clinical indicators (7×7 questionnaire) showed an improvement in the condition of patients who had taken dietary supplements together with conventional therapy.

With regard to microbiome composition, there was no variation observed between groups 1 and 2. Even though several bacterial genera passed the *p* < 0.05 threshold, the number of these genera was comparable between visit 1 and 3. However, among these genera, we noticed *Oscillibacter* was dominant in group 1 and *Veillonella*, *Gemmiger*, and *Collinsella* were dominant in Group 2. Previously, an animal study showed higher *Oscillibacter* prevalence in healthy controls than in patients with IBS [44, 45] and high *Veillonella* prevalence in IBS-C patients [46, 47]. Although *Oscillibacter* can promote fiber degradation by an array of cellulases and produce butyric acid from glucose, ribose, and xylose, the main metabolite produced is valeric acid [48, 49]. Previously, it was shown that the abundance of *Collinsella* was associated with low a fiber diet and obesity [50], type 2 diabetes [51], and atherosclerosis [52]. Lower content of the *Collinsella* family (Coriobacteriaceae) was associated with a healthier microbiome composition [53]. No data exists for *Gemmiger* prevalence in patients with functional disorders or healthy controls.

We noticed slight changes between group 1 and 2 in the number of bacteria containing genes involved in the synthesis of fatty acids. However, these changes were not statistically significant after correcting for FDR and no correlations were observed with symptom severity. In general, an increased number of bacteria implicated in fatty acid biosynthesis could improve intestinal permeability through mucus layer generation [54].

When analyzing associations with the severity of IBS, we found positive correlations with the abundance of *Fusobacterium*. Moreover, the presence/abundance of these bacteria was strongly associated with increased discomfort and pain. Previous reports found that *Fusobacterium* increased severity of diarrhea-predominant IBS (IBS-D) [55], and *Fusobacterium nucleatum* was involved in the pathogenesis of IBS by causing microbial dysbiosis and exacerbating visceral hypersensitivity [55]. The metabolites of *Fusobacterium* contain butyric acid, which promotes visceral hypersensitivity in an IBS-like model via an enteric glial cell-derived nerve growth factor [56]. However, some studies have shown that *Fusobacterium* was decreased in IBS-D patients [57].

The present study revealed negative correlations between *Acidaminococcaceae* and *Enterobacteriaceae* abundance and positive correlations of *Streptococcaceae*, *Coriobacteriaceae*, and *Veillonellaceae* abundance with IBS severity. Except for *Enterobacteriaceae*, all of these families have been associated with symptom severity in IBS patients (especially IBS-D) compared to healthy controls or patients with other gastrointestinal diseases [58–62].

The current study has several limitations. First, the sample size was relatively small and comprised only 56 patients. Further studies with more participants are needed to validate the trends observed. Moreover, we did not perform follow-up assessments to record the long-term treatment effects. This limitation will also be addressed in future trials.

## Conclusion

The components of the food supplement “Standart Zdorovya GASTRO” standardized for menthol, limonene, and gingerol influence the main pathogenetic mechanisms of IBS and FD and increase the efficacy of standard therapy for IBS and FD. The use of the supplement is safe and does not cause side effects.

## Supporting information

S1 File (DOCX)

S2 File (JPEG)

S3 File Supplementary figures. (DOCX)

S4 File Supplementary tables. (PDF)

S1 Checklist CONSORT 2010 checklist of information to include when reporting a randomised trial*. (DOC)

## References

[1] Perveen I, Rahman MM, Saha M, Rahman MM, Hasan MQ. Prevalence of irritable bowel syndrome and functional dyspepsia, overlapping symptoms, and associated factors in a general population of Bangladesh. Indian J Gastroenterol. 2014;33: 265–273. doi: 10.1007/s12664-014-0447-1 24664445

[2] Vakil N, Stelwagon M, Shea EP, Miller S. Symptom burden and consulting behavior in patients with overlapping functional disorders in the US population. United Eur Gastroenterol J. 2016. doi: 10.1177/2050640615600114 27403308 PMC4924424

[3] Lee BJ, Bak YT. Irritable bowel syndrome, gut microbiota and probiotics. J Neurogastroenterol Motil. 2011;17: 252–266. doi: 10.5056/jnm.2011.17.3.252 21860817 PMC3155061

[4] Oka P, Parr H, Barberio B, Black CJ, Savarino E V., Ford AC. Global prevalence of irritable bowel syndrome according to Rome III or IV criteria: a systematic review and meta-analysis. Lancet Gastroenterol Hepatol. 2020. doi: 10.1016/S2468-1253(20)30217-X 32702295

[5] Lacy BE, Mearin F, Chang L, Chey WD, Lembo AJ, Simren M, et al. Bowel disorders. Gastroenterology. 2016;150: 1393–1407. doi: 10.1053/j.gastro.2016.02.031 27144627

[6] Irritable bowel syndrome: A mild disorder; Purely symptomatic treatment. Prescrire Int. 2009;18: 75–79. 19585728

[7] Ford AC, Lacy BE, Harris LA, Quigley EMM, Moayyedi P. Effect of Antidepressants and Psychological Therapies in Irritable Bowel Syndrome. Am J Gastroenterol. 2019;114: 21–39. doi: 10.1038/s41395-018-0222-5 30177784

[8] Kleinstäuber M, Witthöft M, Steffanowski A, van Marwijk H, Hiller W, Lambert MJ. Pharmacological interventions for somatoform disorders in adults. Cochrane Database Syst Rev. 2014; CD010628. doi: 10.1002/14651858.CD010628.pub2 25379990 PMC11023023

[9] Dale HF, Rasmussen SH, Asiller ÖÖ, Lied GA. Probiotics in irritable bowel syndrome: An up-to-date systematic review. Nutrients. 2019;11: 2048. doi: 10.3390/nu11092048 31480656 PMC6769995

[10] Pittayanon R, Lau JT, Yuan Y, Leontiadis GI, Tse F, Surette M, et al. Gut Microbiota in Patients With Irritable Bowel Syndrome—A Systematic Review. Gastroenterology. 2019. doi: 10.1053/j.gastro.2019.03.049 30940523

[11] Morrison DJ, Preston T. Formation of short chain fatty acids by the gut microbiota and their impact on human metabolism. Gut Microbes. 2016;7: 189–200. doi: 10.1080/19490976.2015.1134082 26963409 PMC4939913

[12] Coss-Adame E, Rao SSC. Brain and gut interactions in irritable bowel syndrome: New paradigms and new understandings. Curr Gastroenterol Rep. 2014;16: 1–8. doi: 10.1007/s11894-014-0379-z 24595616 PMC4083372

[13] Simrén M, Törnblom H, Palsson OS, Van Oudenhove L, Whitehead WE, Tack J. Cumulative Effects of Psychologic Distress, Visceral Hypersensitivity, and Abnormal Transit on Patient-reported Outcomes in Irritable Bowel Syndrome. Gastroenterology. 2019;157: 391-402.e2. doi: 10.1053/j.gastro.2019.04.019 31022401

[14] Duan R, Zhu S, Wang B, Duan L. Alterations of gut microbiota in patients with irritable bowel syndrome based on 16s rRNA-targeted sequencing: A systematic review. Clin Transl Gastroenterol. 2019;10: e00012. doi: 10.14309/ctg.0000000000000012 30829919 PMC6407812

[15] Trush EA, Poluektova EA, Beniashvilli AG, Shifrin OS, Poluektov YM, Ivashkin VT. The Evolution of Human Probiotics: Challenges and Prospects. Probiotics and Antimicrobial Proteins. 2020. doi: 10.1007/s12602-019-09628-4 31907861

[16] Didari T, Mozaffari S, Nikfar S, Abdollahi M. Effectiveness of probiotics in irritable bowel syndrome: Updated systematic review with meta-analysis. World J Gastroenterol. 2015;21: 3072–3084. doi: 10.3748/wjg.v21.i10.3072 25780308 PMC4356930

[17] Ford AC, Lacy BE, Talley NJ. Irritable Bowel Syndrome. N Engl J Med. 2017;376: 2566–2578. doi: 10.1056/NEJMra1607547 28657875

[18] Amato A, Liotta R, Mulè F. Effects of menthol on circular smooth muscle of human colon: Analysis of the mechanism of action. Eur J Pharmacol. 2014;740: 295–301. doi: 10.1016/j.ejphar.2014.07.018 25046841

[19] Gaudioso C, Hao J, Martin-Eauclaire MF, Gabriac M, Delmas P. Menthol pain relief through cumulative inactivation of voltage-gated sodium channels. Pain. 2012;153: 473–484. doi: 10.1016/j.pain.2011.11.014 22172548

[20] Pan R, Tian Y, Gao R, Li H, Zhao X, Barrett JE, et al. Central mechanisms of menthol-induced analgesia. J Pharmacol Exp Ther. 2012;343: 661–672. doi: 10.1124/jpet.112.196717 22951274

[21] Imai H, Osawa K, Yasuda H, Hamashima H, Arai T, Sasatsu M. Inhibition by the essential oils of peppermint and spearmint of the growth of pathogenic bacteria. Microbios. 2001;106: 31–39. 11549238

[22] Patrick L. Gastroesophageal reflux disease (GERD): a review of conventional and alternative treatments. Altern Med Rev. 2011;16: 116–133. Available: http://search.ebscohost.com/login.aspx?direct=true&profile=ehost&scope=site&authtype=crawler&jrnl=10895159&AN=61445441&h=Po9FKCHAth5IC0RATltSqXC3FKSxDGA3LB%2FxS1zJR75kiTBjsKt3NhESxGtQabGE36UGKZ4E8h3b0iq4HLGYWg%3D%3D&crl=c 21649454

[23] D’Alessio PA, Ostan R, Bisson JF, Schulzke JD, Ursini M V., Béné MC. Oral administration of d-Limonene controls inflammation in rat colitis and displays anti-inflammatory properties as diet supplementation in humans. Life Sci. 2013;92: 1151–1156. doi: 10.1016/j.lfs.2013.04.013 23665426

[24] Rehman MU, Tahir M, Khan AQ, Khan R, Oday-O-Hamiza, Lateef A, et al. d-limonene suppresses doxorubicin-induced oxidative stress and inflammation via repression of COX-2, iNOS, and NFκB in kidneys of Wistar rats. Exp Biol Med. 2014;239: 465–476. doi: 10.1177/1535370213520112 24586096

[25] Ghayur MN, Gilani AH. Pharmacological basis for the medicinal use of ginger in gastrointestinal disorders. Dig Dis Sci. 2005;50: 1889–1897. doi: 10.1007/s10620-005-2957-2 16187193

[26] Giacosa A, Morazzoni P, Bombardelli E, Riva A, Porro GB, Rondanelli M. Can nausea and vomiting be treated with Ginger extract? Eur Rev Med Pharmacol Sci. 2015;19: 1291–1296. 25912592

[27] Simon A, Darcsi A, Kéry Á, Riethmüller E. Blood-brain barrier permeability study of ginger constituents. J Pharm Biomed Anal. 2020;177: 112820. doi: 10.1016/j.jpba.2019.112820 31476432

[28] Ivashkin VT, Shelygin Y., Baranskaya YK, Belousova YA, Beniashvili AG, Vasilyev S., et al. Diagnosis and treatment of the irritable bowel syndrome: clinical guidelines of the Russian gastroenterological association and Russian association of coloproctology. gastro-j.ru. 2017;27: 76–93. 10.22416/1382-4376-2017-27-5-76-93

[29] Ivashkin VT, Mayev IV, Sheptulin AA, Lapina TL, Trukhmanov AS, Kartavenko IM, et al. Diagnosis and treatment of the functional dyspepsia: clinical guidelines of the Russian Gastroenterological Association. Ross Z Gastroenterol Gepatol Koloproktol. 2017;27: 50–61. 10.22416/1382-4376-2017-27-1-50-61

[30] Faul F, Erdfelder E, Lang AG, Buchner A. G*Power 3: A flexible statistical power analysis program for the social, behavioral, and biomedical sciences. Behavior Research Methods. 2007. doi: 10.3758/BF03193146 17695343

[31] Ivashkin VT, Poluektova EA, Glazunov AB, Putilovskiy MA, Epstein OI. Pathogenetic approach to the treatment of functional disorders of the gastrointestinal tract and their intersection: Results of the Russian observation retrospective program COMFORT. BMC Gastroenterol. 2019. doi: 10.1186/s12876-019-1143-5 31892312 PMC6938622

[32] Ivashkin V, Sheptulin A, Shifrin O, Poluektova E, Pavlov C, Ivashkin K, et al. Clinical validation of the “7 × 7” questionnaire for patients with functional gastrointestinal disorders. J Gastroenterol Hepatol. 2019;34: 1042–1048. doi: 10.1111/jgh.14546 30462850

[33] Parikh HI, Koparde VN, Bradley SP, Buck GA, Sheth NU. MeFiT: Merging and filtering tool for illumina paired-end reads for 16S rRNA amplicon sequencing. BMC Bioinformatics. 2016;17: 491. doi: 10.1186/s12859-016-1358-1 27905885 PMC5134250

[34] Callahan BJ, McMurdie PJ, Rosen MJ, Han AW, Johnson AJA, Holmes SP. DADA2: High-resolution sample inference from Illumina amplicon data. Nat Methods. 2016;13: 581–583. doi: 10.1038/nmeth.3869 27214047 PMC4927377

[35] Cole JR, Wang Q, Fish JA, Chai B, McGarrell DM, Sun Y, et al. Ribosomal Database Project: Data and tools for high throughput rRNA analysis. Nucleic Acids Res. 2014;42: D633–D642. doi: 10.1093/nar/gkt1244 24288368 PMC3965039

[36] Narayan NR, Weinmaier T, Laserna-Mendieta EJ, Claesson MJ, Shanahan F, Dabbagh K, et al. Piphillin predicts metagenomic composition and dynamics from DADA2-corrected 16S rDNA sequences. BMC Genomics. 2020;21: 56. doi: 10.1186/s12864-019-6427-1 31952477 PMC6967091

[37] R Development Core Team. R: A language and environment for statistical computing. Vienna, Austria. 2017. doi:R Foundation for Statistical Computing, Vienna, Austria. ISBN 3-900051-07-0, URL http://www.R-project.org.

[38] Wickham H. ggplot2: Elegant Graphics for Data Analysis. Second Edition. Springer. Media. 2016. doi: 10.1007/978-0-387-98141-3

[39] Sarkar A, Lehto SM, Harty S, Dinan TG, Cryan JF, Burnet PWJ. Psychobiotics and the Manipulation of Bacteria–Gut–Brain Signals. Trends Neurosci. 2016;39: 763–781. doi: 10.1016/j.tins.2016.09.002 27793434 PMC5102282

[40] König J, Wells J, Cani PD, García-Ródenas CL, MacDonald T, Mercenier A, et al. Human intestinal barrier function in health and disease. Clin Transl Gastroenterol. 2016;7: e196. doi: 10.1038/ctg.2016.54 27763627 PMC5288588

[41] Ivashkin VT, Mayev IV, Ivashkin KV, Korochanskaya NV, Lopina OD, Lapina TL, et al. The role of protective factors disorders of acid-related diseases pathogenesis. Ross Z Gastroenterol Gepatol Koloproktol. 2016;26: 115–116. 10.22416/1382-4376-2016-26-3

[42] Hijova E, Chmelarova A. Short chain fatty acids and colonic health. Bratisl Lek Listy. 2007;108: 354–358. 18203540

[43] Louis P, Hold GL, Flint HJ. The gut microbiota, bacterial metabolites and colorectal cancer. Nat Rev Microbiol. 2014;12: 661–672. doi: 10.1038/nrmicro3344 25198138

[44] Tap J, Derrien M, Törnblom H, Brazeilles R, Cools-Portier S, Doré J, et al. Identification of an Intestinal Microbiota Signature Associated With Severity of Irritable Bowel Syndrome. Gastroenterology. 2017;152: 111–123.e8. doi: 10.1053/j.gastro.2016.09.049 27725146

[45] Wang X, Qi Q, Wang Y, Wu H, Jin X, Yao H, et al. Gut microbiota was modulated by moxibustion stimulation in rats with irritable bowel syndrome. Chin Med. 2018;13: 63. doi: 10.1186/s13020-018-0220-y 30574173 PMC6299671

[46] Tana C, Umesaki Y, Imaoka A, Handa T, Kanazawa M, Fukudo S. Altered profiles of intestinal microbiota and organic acids may be the origin of symptoms in irritable bowel syndrome. Neurogastroenterol Motil. 2010;22: 512–e115. doi: 10.1111/j.1365-2982.2009.01427.x 19903265

[47] Lee KJ, Tack J. Altered intestinal microbiota in irritable bowel syndrome. Neurogastroenterol Motil. 2010;22: 493–498. doi: 10.1111/j.1365-2982.2010.01482.x 20414959

[48] Lee GH, Kumar S, Lee JH, Chang DH, Kim DS, Choi SH, et al. Genome sequence of oscillibacter ruminantium strain GH1, isolated from rumen of korean native cattle. J Bacteriol. 2012;194: 6362. doi: 10.1128/JB.01677-12 23105088 PMC3486395

[49] Katano Y, Fujinami S, Kawakoshi A, Nakazawa H, Oji S, Iino T, et al. Complete genome sequence of Oscillibacter valericigenes Sjm18-20 T (= NBRC 101213 T). Stand Genomic Sci. 2012;6: 406–414. doi: 10.4056/sigs.2826118 23408234 PMC3558957

[50] Gomez-Arango LF, Barrett HL, Wilkinson SA, Callaway LK, McIntyre HD, Morrison M, et al. Low dietary fiber intake increases Collinsella abundance in the gut microbiota of overweight and obese pregnant women. Gut Microbes. 2018;9: 189–201. doi: 10.1080/19490976.2017.1406584 29144833 PMC6219589

[51] Lambeth SM, Carson T, Lowe J, Ramaraj T, Leff JW, Luo L, et al. Composition Diversity and Abundance of Gut Microbiome in Prediabetes and Type 2 Diabetes. J Diabetes Obes. 2015;2: 1–7. doi: 10.15436/2376-0949.15.031 26756039 PMC4705851

[52] Karlsson FH, Fåk F, Nookaew I, Tremaroli V, Fagerberg B, Petranovic D, et al. Symptomatic atherosclerosis is associated with an altered gut metagenome. Nat Commun. 2012;3. doi: 10.1038/ncomms2266 23212374 PMC3538954

[53] Astbury S, Atallah E, Vijay A, Aithal GP, Grove JI, Valdes AM. Lower gut microbiome diversity and higher abundance of proinflammatory genus Collinsella are associated with biopsy-proven nonalcoholic steatohepatitis. Gut Microbes. 2019;11: 569–580. doi: 10.1080/19490976.2019.1681861 31696774 PMC7524262

[54] Mariadason JM, Barkla DH, Gibson PR. Effect of short-chain fatty acids on paracellular permeability in Caco- 2 intestinal epithelium model. Am J Physiol—Gastrointest Liver Physiol. 1997;272: G705–G712. doi: 10.1152/ajpgi.1997.272.4.G705 9142899

[55] Gu X, Song LJ, Li LX, Liu T, Zhang MM, Li Z, et al. Fusobacterium nucleatum Causes Microbial Dysbiosis and Exacerbates Visceral Hypersensitivity in a Colonization-Independent Manner. Front Microbiol. 2020. doi: 10.3389/fmicb.2020.01281 32733392 PMC7358639

[56] Long X, Li M, Li LX, Sun YY, Zhang WX, Zhao DY, et al. Butyrate promotes visceral hypersensitivity in an IBS-like model via enteric glial cell-derived nerve growth factor. Neurogastroenterol Motil. 2018. doi: 10.1111/nmo.13227 29052293

[57] Mei L, Zhou J, Su Y, Mao K, Wu J, Zhu C, et al. Gut microbiota composition and functional prediction in diarrhea-predominant irritable bowel syndrome. BMC Gastroenterol. 2021. doi: 10.1186/s12876-021-01693-w 33663411 PMC7934555

[58] Carco C, Young W, Gearry RB, Talley NJ, McNabb WC, Roy NC. Increasing Evidence That Irritable Bowel Syndrome and Functional Gastrointestinal Disorders Have a Microbial Pathogenesis. Frontiers in Cellular and Infection Microbiology. 2020. doi: 10.3389/fcimb.2020.00468 33014892 PMC7509092

[59] Barandouzi ZA, Lee J, Maas K, Starkweather AR, Cong XS. Altered gut microbiota in irritable bowel syndrome and its association with food components. J Pers Med. 2021. doi: 10.3390/jpm11010035 33429936 PMC7827153

[60] Durbán A, Abellán JJ, Jiménez-Hernández N, Artacho A, Garrigues V, Ortiz V, et al. Instability of the faecal microbiota in diarrhoea-predominant irritable bowel syndrome. FEMS Microbiol Ecol. 2013. doi: 10.1111/1574-6941.12184 23889283

[61] Si JM, Yu YC, Fan YJ, Chen SJ. Intestinal microecology and quality of life in irritable bowel syndrome patients. World J Gastroenterol. 2004. doi: 10.3748/wjg.v10.i12.1802 15188510 PMC4572273

[62] Liu Y, Yuan X, Li L, Lin L, Zuo X, Cong Y, et al. Increased Ileal Immunoglobulin A Production and Immunoglobulin A-Coated Bacteria in Diarrhea-Predominant Irritable Bowel Syndrome. Clin Transl Gastroenterol. 2020. doi: 10.14309/ctg.0000000000000146 32352710 PMC7145038

