# Supplementary material for: Efficacy and safety of a food supplement with standardized menthol, limonene, and gingerol content in patients with irritable bowel syndrome: A double-blind, randomized, placebo-controlled trial
Source: PLoS One. 2022 Jun 15;17(6):e0263880. doi: 10.1371/journal.pone.0263880 (PMC9200470; doi:10.1371/journal.pone.0263880)
Supplement: S1 File — (DOCX) [file pone.0263880.s001.docx]

| 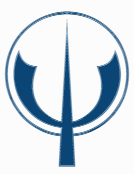 | **FEDERAL STATE BUDGETARY SCIENTIFIC INSTITUTION**  **MENTAL HEALTH RESEARCH CENTER**  **(FSBSI MHRC)** | | |
| --- | --- | --- | --- |
| 115522, Москва, Каширское шоссе 34  [www.psychiatry.ru](http://www.psychiatry.ru) [www.ncpz.ru](http://www.ncpz.ru)  [ncpz@ncpz.ru](mailto:ncpz@ncpz.ru) | | Tel. +7(495)109-03-93  Fax +7(495)109-03-67 | 34, Kashirskoye Sh.  Moscow, 115522, Russia |

**CONCLUSION**

**of FSBI MHRC ETHICS COMMITTEE**

The Ethics Committee of the FSBSI MHRC at its meeting on January 31^st^, 2018 (report No. 418) reviewed **approval** of changes made in the text of the Patient Informed Consent to participate in a scientific study according to the trial protocol "Double-blind, placebo-controlled study of the efficacy of the nutraceutical product "Standart Zdorovya GASTRO" in patients with irritable bowel syndrome, an interdisciplinary study"

**Principal Investigator -** Doctor of Medical Sciences, Professor, Head of psychopharmacological laboratory M.A. Morozova.

**Co-investigators:** Candidate of Medical Sciences, Senior Researcher A.G. Beniashvili; Candidate of Psychological Sciences, Senior Researcher G.E. Rupchev; clinical psychologist A.A. Alekseev.

The members of the ethics committee of the FSBSI MHRC examined the documentation submitted for their review:

1. Patient Information Leaflet and Informed Consent Form of the patient taking part in the study.

The Ethics Committee of the FSBSI MHRC decided to **approve** the changes made in the text of the Patient Informed Consent to participate in a scientific study according to the trial protocol "Double-blind, placebo-controlled study of the efficacy of the nutraceutical product "Standart Zdorovya GASTRO" in patients with irritable bowel syndrome, an interdisciplinary study"

The list of ethics committee members is attached.

| FSBSI MHRC Ethics Committee Chair,  professor | Kolyutskaya E.V. |
| --- | --- |
| FSBSI MHRC Ethics Committee Secretary | Pishchik I.N. |
