# Supplementary figures and images for: Efficacy and safety of a food supplement with standardized menthol, limonene, and gingerol content in patients with irritable bowel syndrome: A double-blind, randomized, placebo-controlled trial
Source: PLoS One. 2022 Jun 15;17(6):e0263880. doi: 10.1371/journal.pone.0263880 (PMC9200470; doi:10.1371/journal.pone.0263880)

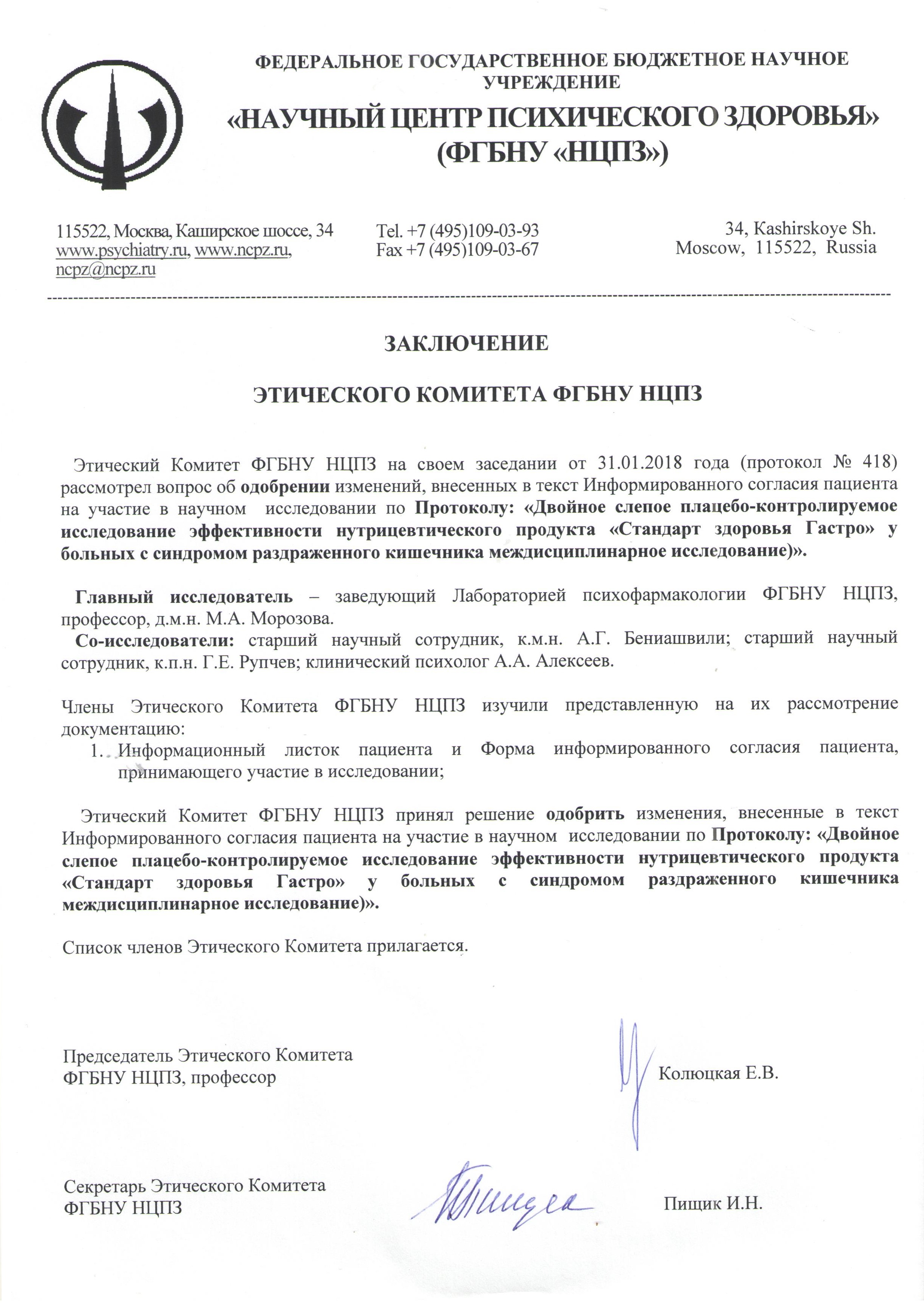

Supplement: S2 File — (JPEG) [file pone.0263880.s002.jpeg]
