# Supplementary material for: Efficacy and safety of a food supplement with standardized menthol, limonene, and gingerol content in patients with irritable bowel syndrome: A double-blind, randomized, placebo-controlled trial
Source: PLoS One. 2022 Jun 15;17(6):e0263880. doi: 10.1371/journal.pone.0263880 (PMC9200470; doi:10.1371/journal.pone.0263880)
Supplement: S3 File — (DOCX) [file pone.0263880.s003.docx]

**S3 File**

**
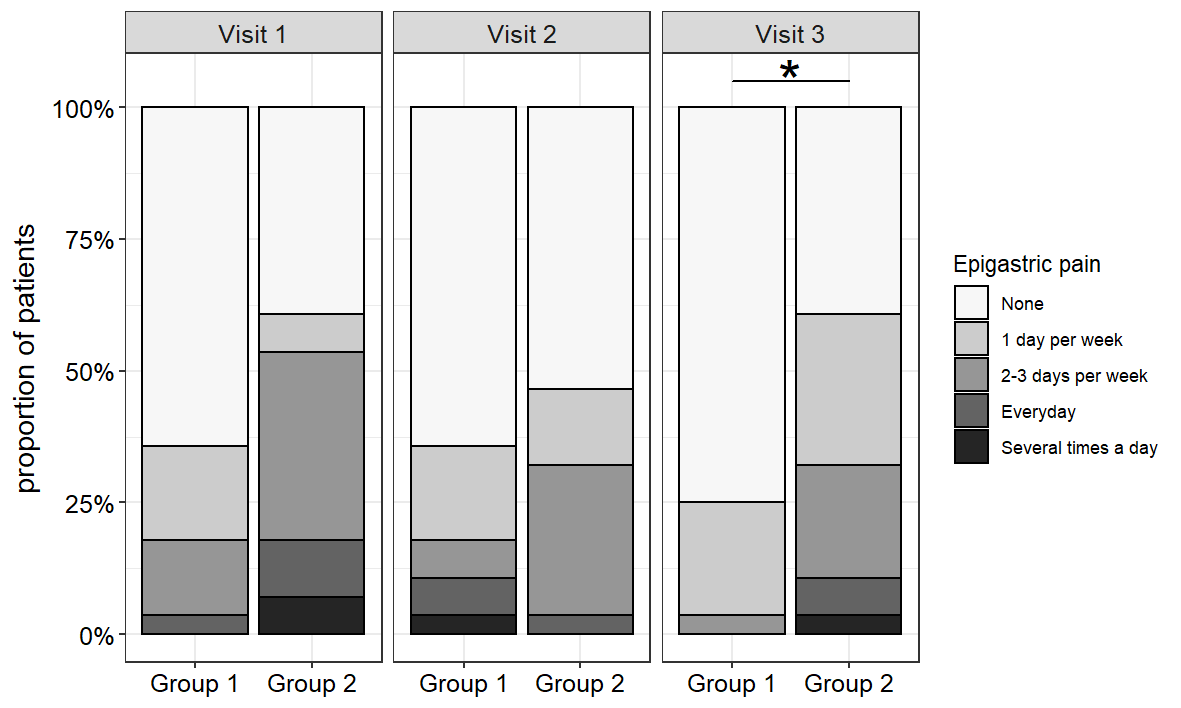
**

**S1 Fig. Number of patients suffering from epigastric pain in Groups 1 and 2 on Visits 1,2 and 3.** *****p <0.05 - The statistical significant difference. There was no statistically significant difference between the number of patients suffering from epigastric pain on Visits 1 and 2. At Visit 3 the number of these patients was significantly lower in Group 1 in compare to Group 2 (p <0.05).


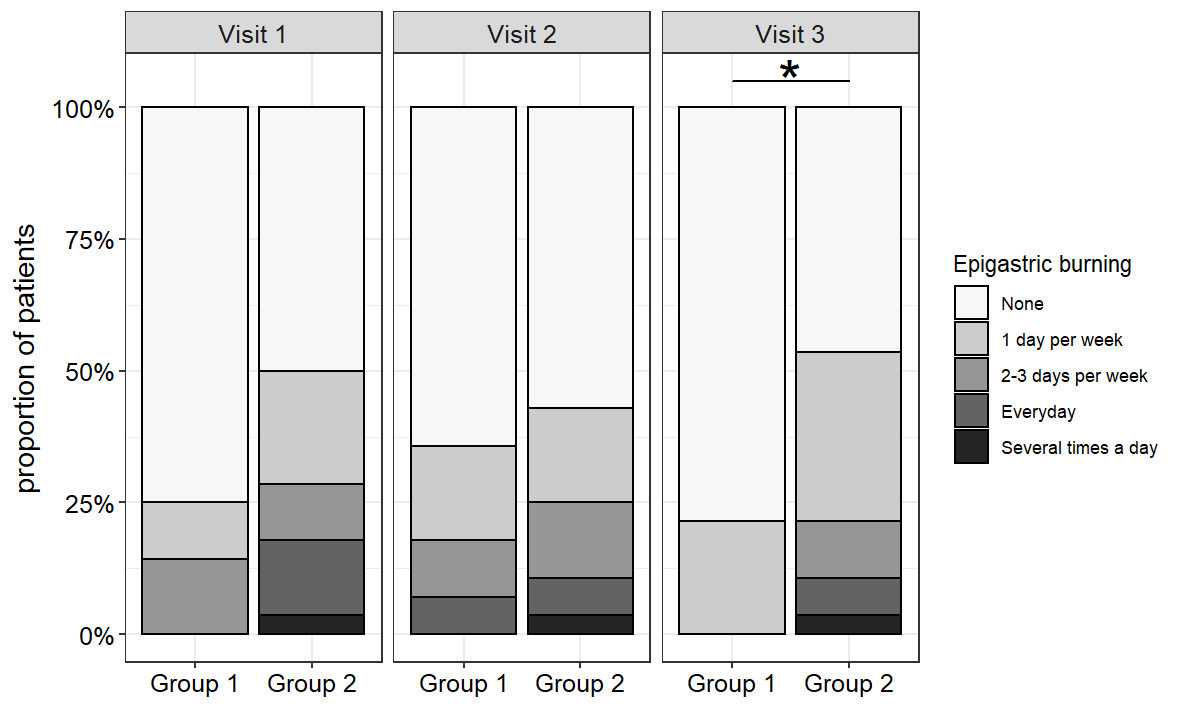


**S2 Fig. Number of patients suffering from epigastric burning in Groups 1 and 2 on Visits 1,2 and 3.** *****p <0.05 - The statistical significant difference. There was no statistically significant difference between the number of patients suffering from epigastric pain on Visits 1 and 2. At Visit 3 the number of these patients was significantly lower in Group 1 in compare to Group 2 (p <0.05).


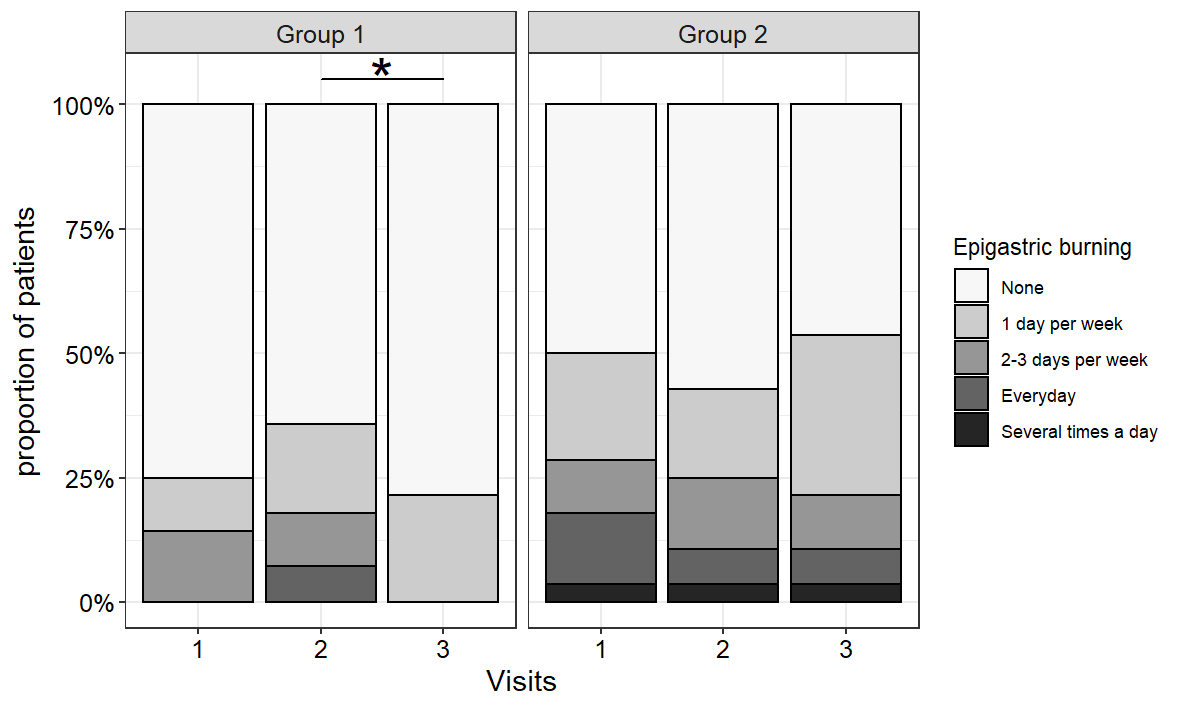


**S3 Fig. Decrease in the number of patients suffering from epigastric burning in Groups 1 and 2 on Visits 1,2 and 3.** *****p <0.05 - The statistical significant difference. Epigastric burning was identified in 25%, 36%, 21% of Group 1 patients and in 50%, 43% and 54% of the Group 2 patients on Visits 1,2 and 3 respectively. A statistically significant difference in the decreasing of epigastric burning was found only in patients Groups 1 from Visits 2 to Visit 3.


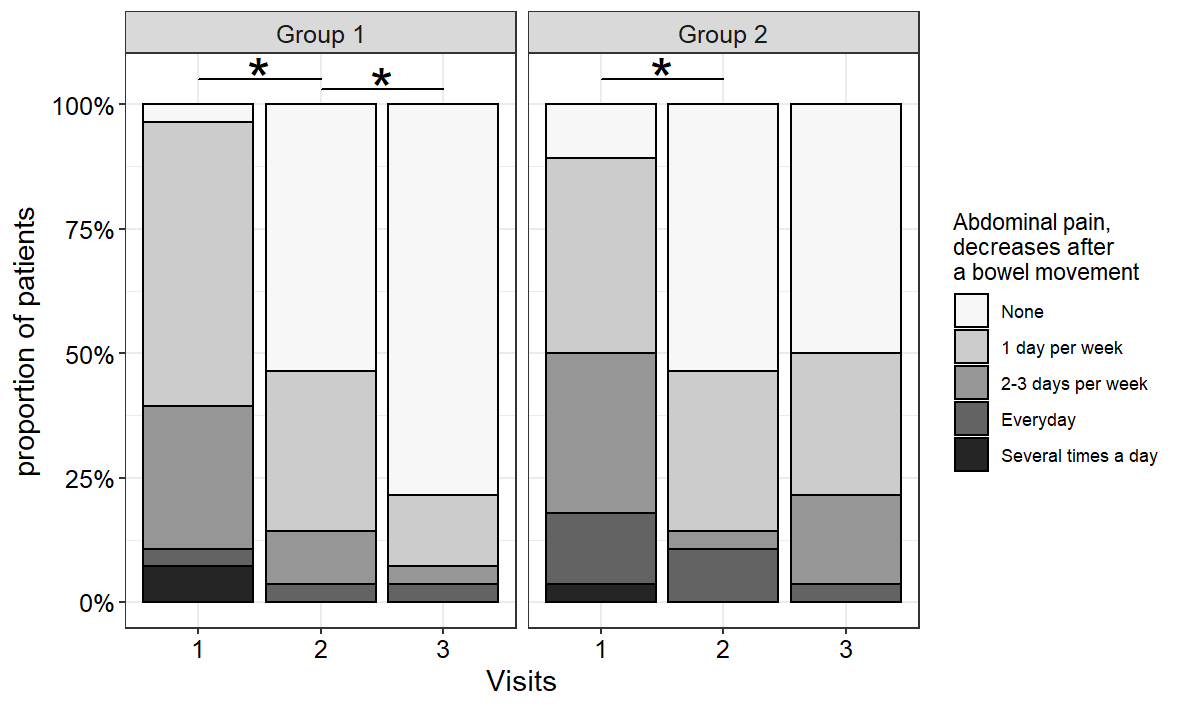


**S4 Fig. Declining the number of patients suffering from abdominal pain, decreases after a bowel movement in Groups 1 and 2 on Visits 1,2 and 3.** *****p <0.05 - The statistical significant difference. Abdominal pain, decreases after a bowel movement was identified in 96%, 46%, 21% of Group 1 patients and 89%, 46% and 50% of Group 2 patients on Visits 1, 2 and 3 respectively. A statistically significant difference in the number of patients suffering from this symptom was found in Group 1 between Visits 1, 2 and 3; and in Group 2 between Visits 1 and 2 only.


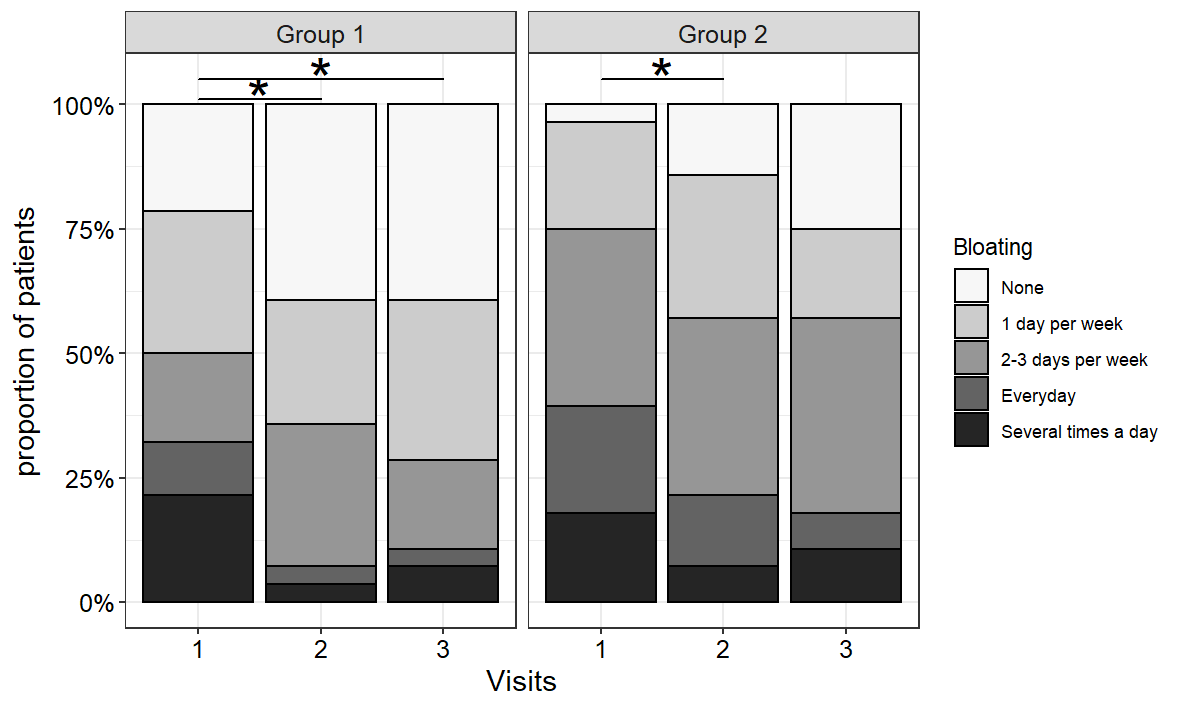


**S5 Fig. Decrease the number of patients suffering from bloating in Groups 1 and 2 on Visits 1,2 and 3.** *****p <0.05 - The statistical significant difference. Bloating was identified in 79%, 61%, 61% of patients of Group 1patients; and in 96%, 76%, 75% of Group 2 patients on Visits 1, 2 and 3 respectively. A statistically significant difference in the decreasing of this symptom was in Group 1 between Visits 1 and 2; 1 and 3; in Group 2 between Visits 1 and 2 only.


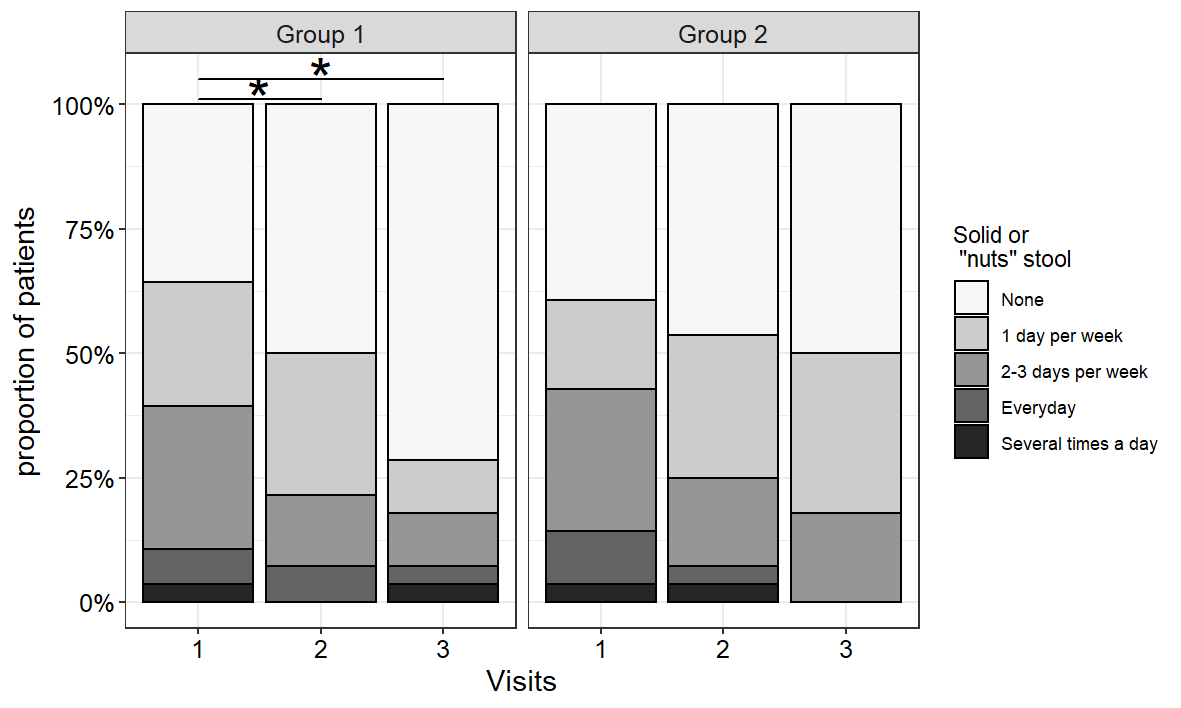


**S6 Fig. Decrease the number of patients suffering from solid or «nuts» stool in Groups 1 and 2 on Visits 1,2 and 3.** *****p <0.05 - The statistical significant difference. Solid or «nuts» stool was identified in 64%, 50%, 29% of Group 1 patients and 61%, 54%, 50% of Group 2 patients on Visits 1,2 and 3 respectively. A statistically significant difference in the decreasing of this symptom was in Group 1 only between Visits 1 and 2 and between Visits 1 and 3.


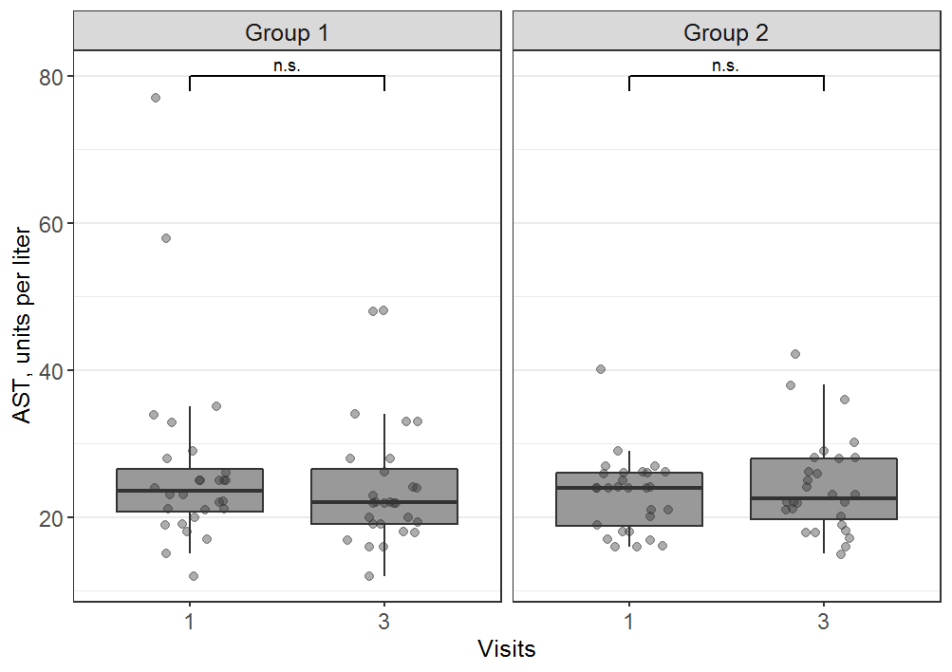
 **S7 Figure.** Comparison of patients in Groups 1 and 2 by Аspartate aminotransferase level (0-35 units per liter) at Visits 1 and 3.


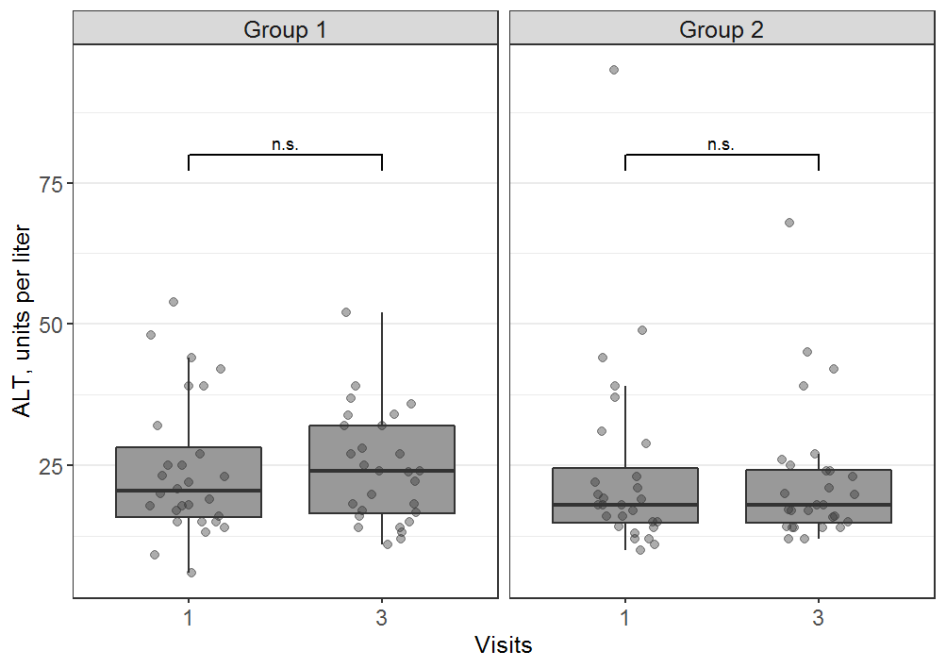
 **S8 Figure.** Comparison of patients in Groups 1 and 2 by Аlanine aminotransferase level (10-40 units per liter) at Visits 1 and 3.


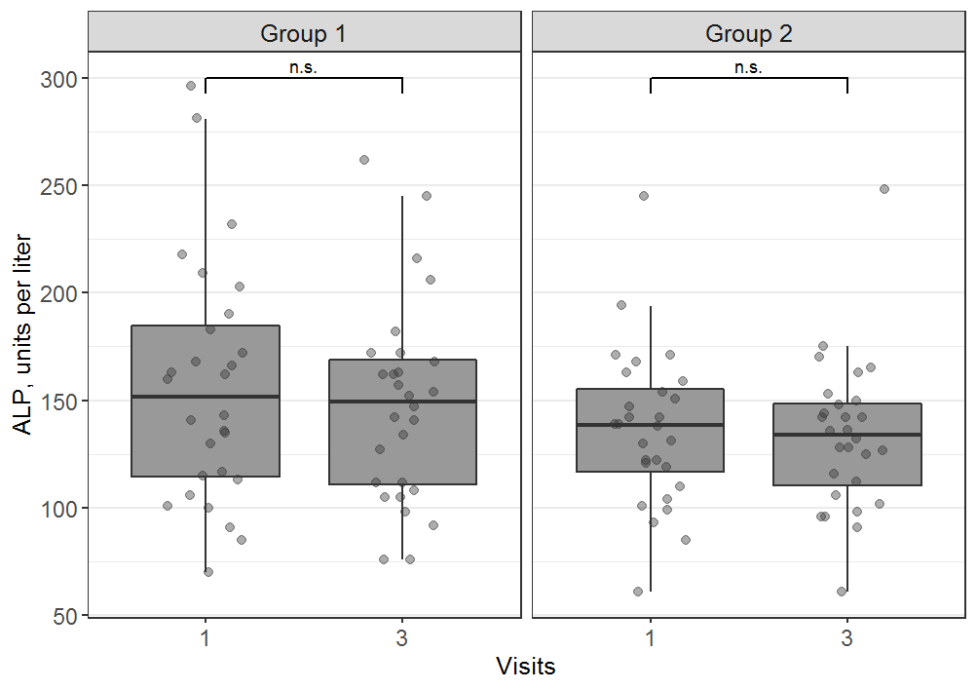
 **S9 Figure.** Comparison of patients in Groups 1 and 2 by Аlkaline phosphatase level (70-290 units per liter) at Visits 1 and 3.


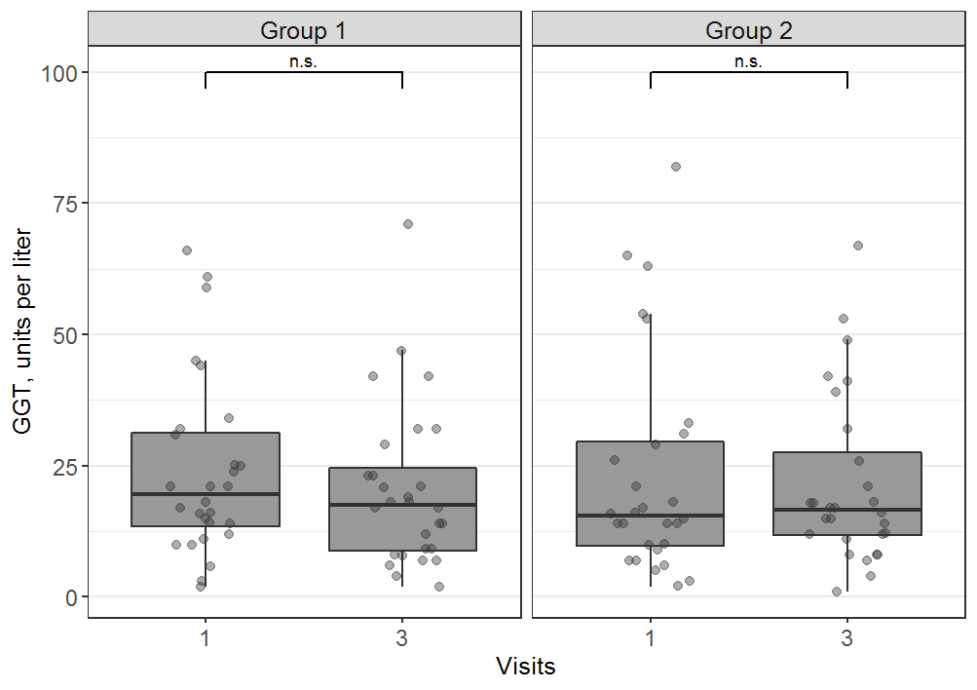
 **S10 Figure.** Comparison of patients in Groups 1 and 2 by Gamma-glutamyltransferase level (0-38 units per liter) at Visits 1 and 3.


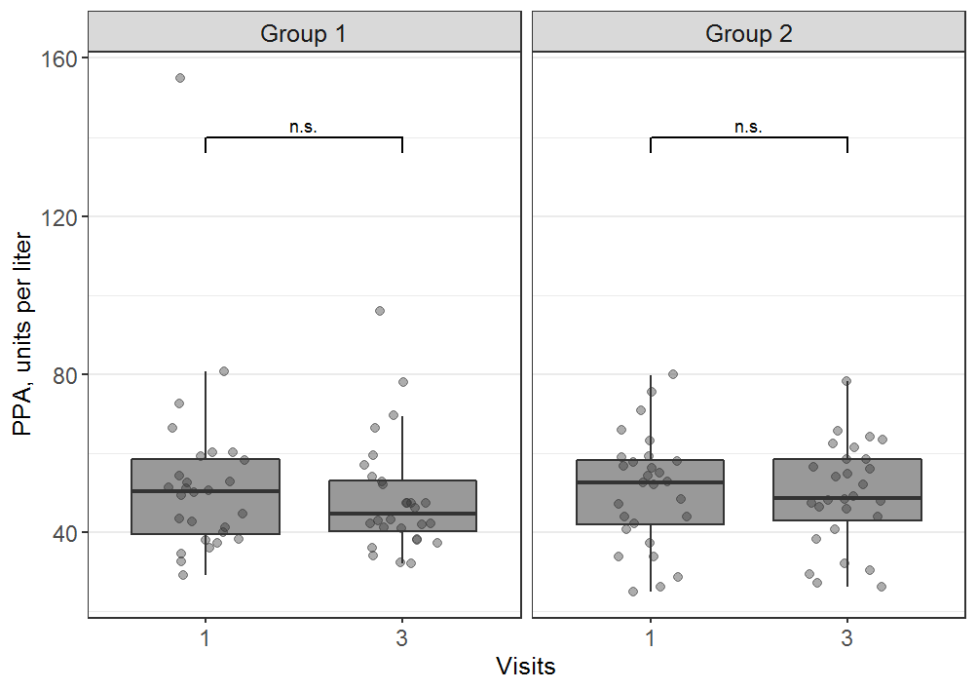
 **S11 Figure.** Comparison of patients in Groups 1 and 2 by Pancreatic alpha-amylase level (25-125 units) at Visits 1 and 3.

**
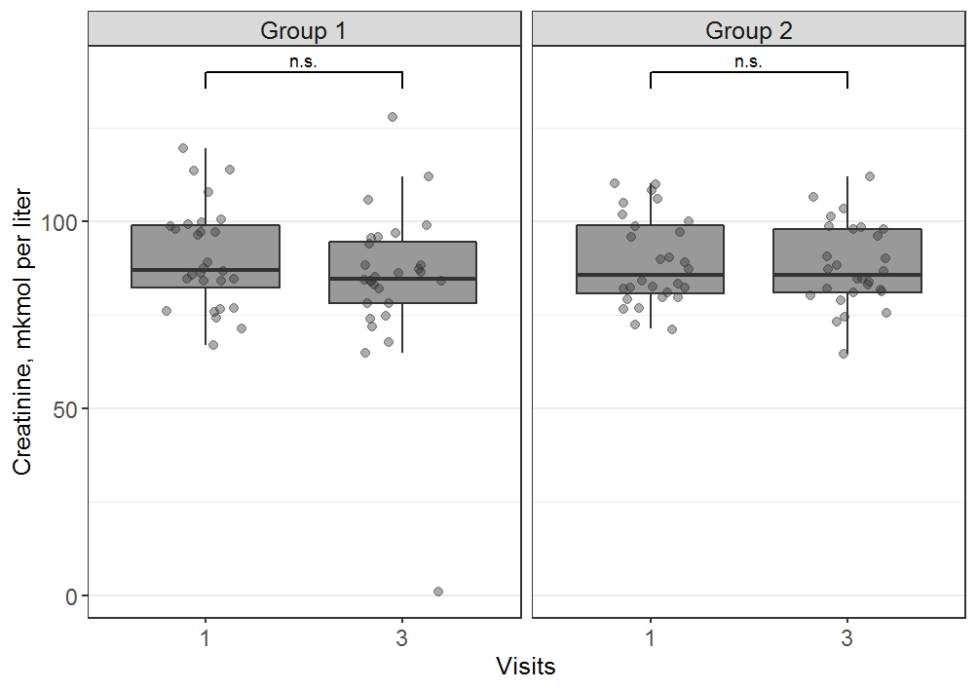
 S12 Figure.** Comparison of patients in Groups 1 and 2 by Creatinine level (44-97 µmol/l) at Visits 1 and 3.

**
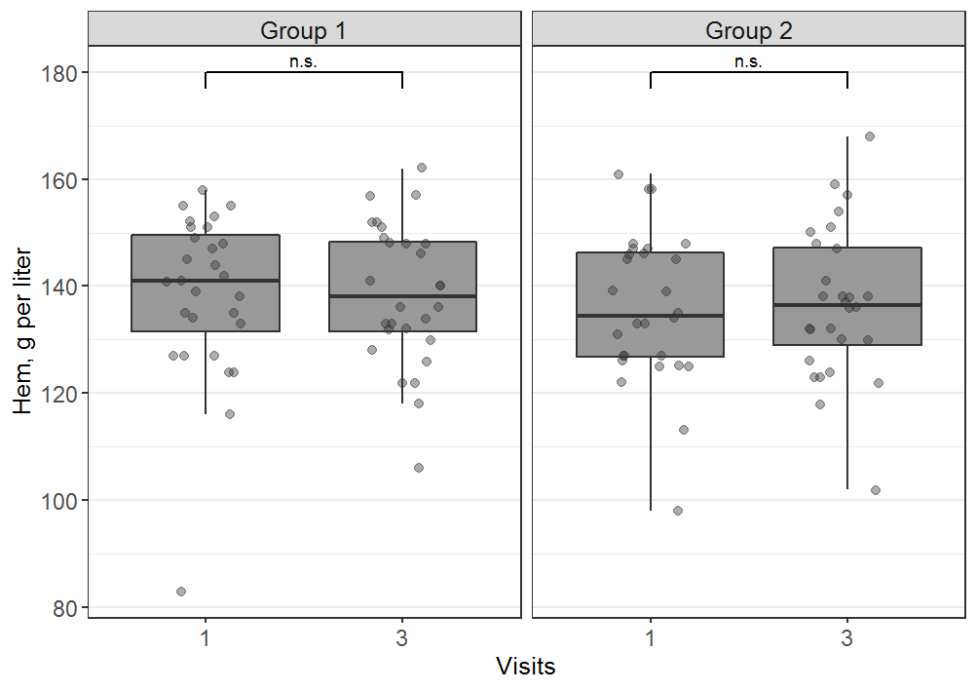
S13 Figure.** Comparison of patients in Groups 1 and 2 by Hemoglobin level (117-160 g per liter) at Visits 1 and 3.

**
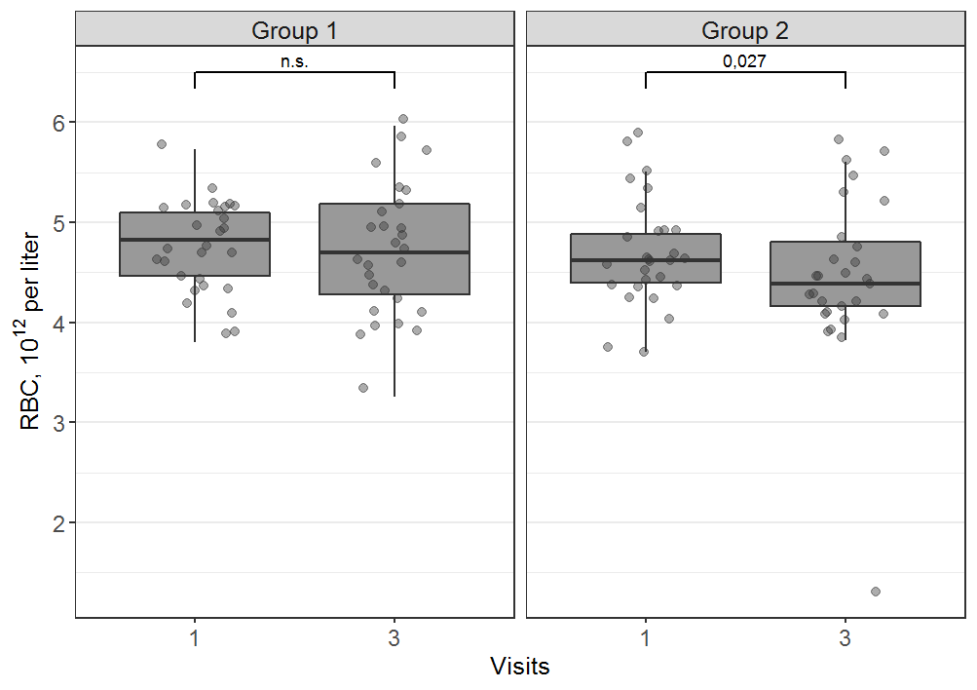
S14 Figure.** Comparison of patients in Groups 1 and 2 by Red Blood Cells count (3,8-5,7x 10^12^ per liter) at Visits 1 and 3.

**
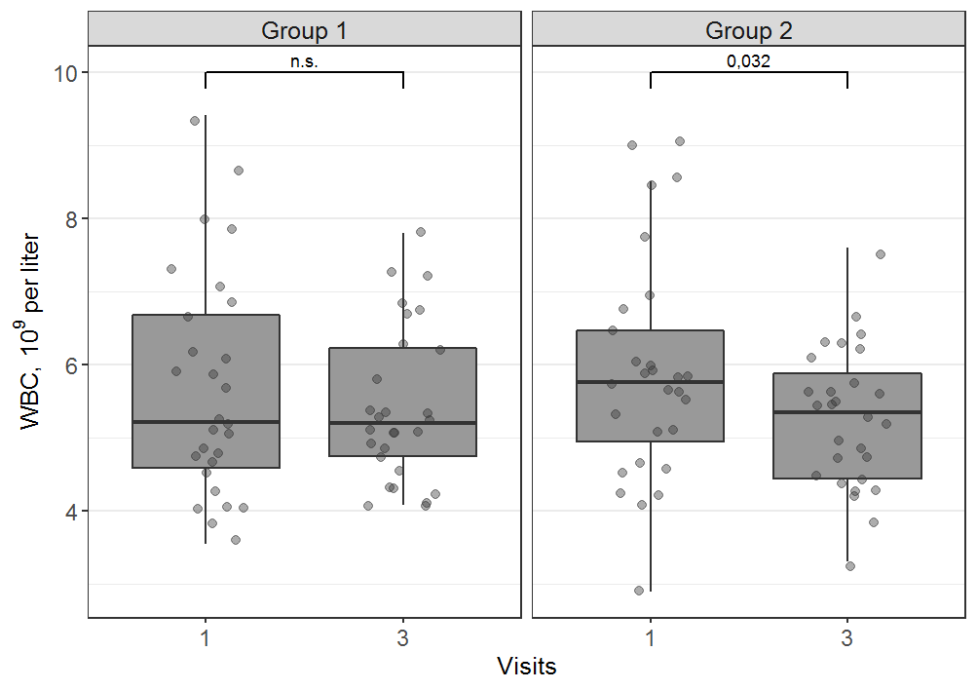
S15 Figure.** Comparison of patients in Groups 1 and 2 by White Blood Cells count (4-11х10^9^ per liter) at Visits 1 and 3.


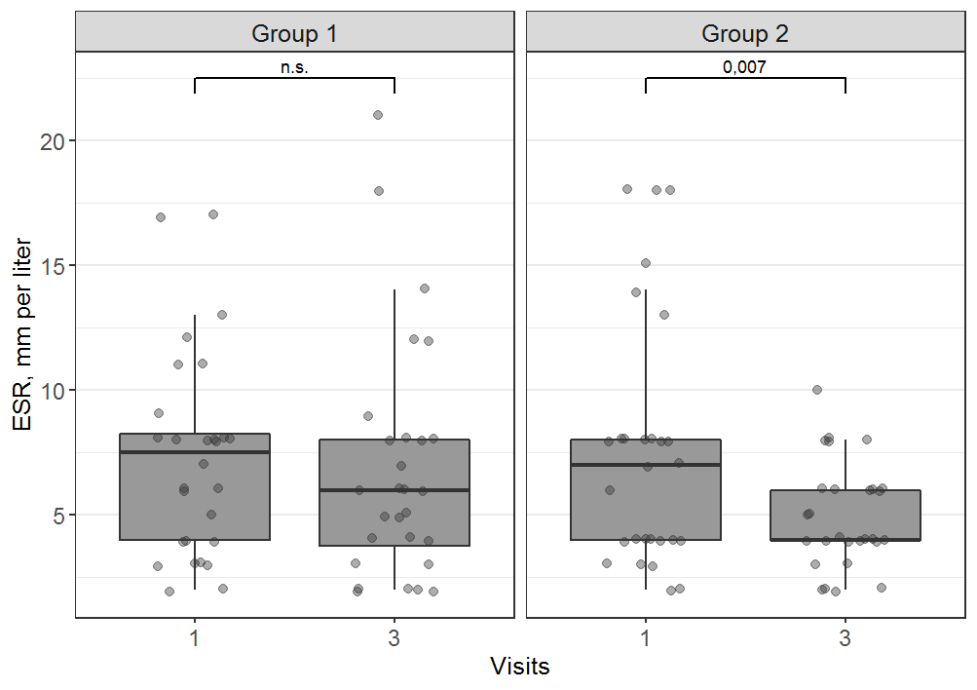
**S16 Figure.** Comparison of patients in Groups 1 and 2 by Erythrocyte Sedimentation Rate (mm/h) at Visits 1 and 3.
