# Supplementary material for: Efficacy and safety of a food supplement with standardized menthol, limonene, and gingerol content in patients with irritable bowel syndrome: A double-blind, randomized, placebo-controlled trial
Source: PLoS One. 2022 Jun 15;17(6):e0263880. doi: 10.1371/journal.pone.0263880 (PMC9200470; doi:10.1371/journal.pone.0263880)
Supplement: S4 File — (PDF) [file pone.0263880.s004.pdf]

Table 1. Friedman rank sum test for total score of the «7×7» questionnaire

Group	n	statistic	df	p	effect size (95% CI)*	magnitude
Group 1	28	26	2	0.0000023	0.46 (0.25-0.73)	moderate
Group 2	28	25.56	2	0.0000028	0.46 (0.25-0.68)	moderate

*- Friedman test effect size (Kendall's W value)

Table 2. Post-hoc analysis of total score of the «7×7» questionnaire using Wilcoxon signed rank test on paired samples

Group	Visits		n	statistic	p	p.adj*	p.adj.signif
Group 1	1	2	28	360.5	0.000343	0.001000	**
	1	3	28	360.5	0.000039	0.000117	***
	2	3	28	292.0	0.003000	0.009000	**
Group 2	1	2	28	348.5	0.0001280	0.000384	***
	1	3	28	374.5	0.0000965	0.000290	***
	2	3	28	184.5	0.8290000	1.000000	ns

*- p-value adjusted by Bonferroni correction
